# Analysis of regulatory protease sequences identified through bioinformatic data mining of the *Schistosoma mansoni *genome

**DOI:** 10.1186/1471-2164-10-488

**Published:** 2009-10-21

**Authors:** David H Bos, Chris Mayfield, Dennis J Minchella

**Affiliations:** 1 Department of Biological Sciences, Purdue University, 915 W State St, West Lafayette, IN 47907, USA; 2 Department of Computer Science, Purdue University, 305 N University St, West Lafayette, IN 47907, USA

## Abstract

**Background:**

New chemotherapeutic agents against *Schistosoma mansoni*, an etiological agent of human schistosomiasis, are a priority due to the emerging drug resistance and the inability of current drug treatments to prevent reinfection. Proteases have been under scrutiny as targets of immunological or chemotherapeutic anti-*Schistosoma *agents because of their vital role in many stages of the parasitic life cycle. Function has been established for only a handful of identified *S. mansoni *proteases, and the vast majority of these are the digestive proteases; very few of the conserved classes of regulatory proteases have been identified from *Schistosoma *species, despite their vital role in numerous cellular processes. To that end, we identified protease protein coding genes from the *S. mansoni *genome project and EST library.

**Results:**

We identified 255 protease sequences from five catalytic classes using predicted proteins of the *S. mansoni *genome. The vast majority of these show significant similarity to proteins in KEGG and the Conserved Domain Database. Proteases include calpains, caspases, cytosolic and mitochondrial signal peptidases, proteases that interact with ubiquitin and ubiquitin-like molecules, and proteases that perform regulated intramembrane proteolysis. Comparative analysis of classes of important regulatory proteases find conserved active site domains, and where appropriate, signal peptides and transmembrane helices. Phylogenetic analysis provides support for inferring functional divergence among regulatory aspartic, cysteine, and serine proteases.

**Conclusion:**

Numerous proteases are identified for the first time in *S. mansoni*. We characterized important regulatory proteases and focus analysis on these proteases to complement the growing knowledge base of digestive proteases. This work provides a foundation for expanding knowledge of proteases in *Schistosoma *species and examining their diverse function and potential as targets for new chemotherapies.

## Background

Schistosomiasis is a common parasitic disease, affecting millions of people, mostly in tropical, developing countries [1]. A causative agent of the disease is a trematode worm, *Schistosoma mansoni*. Treatment of schistosomiasis is commonly accomplished with praziquantel, for which the mechanism of action is not precisely defined but is thought to affect calcium ion channels [2] and/or purine nucleotide uptake [3]. Despite the effectiveness of treatment, reinfection is common, and even more troubling, strains of *S. mansoni *resistant to praziquantel have been found [4]. Thus, additional chemotherapeutic agents and an effective vaccine against this parasite have long been desired [5].

Ideally, vaccination of at-risk populations against the debilitating effects of schistosomiasis is desired, but no such treatment option is currently available. Surface receptors and other proteins are currently being tested for their potential to act as vaccines, but numerous challenges in the search for an effective vaccine have yet to be overcome [6]. Some candidate vaccines are effective agents but cannot be mass produced. Most other candidate proteins have little potential as a vaccine, providing only 40-50% protection [6]. While the search for an effective vaccine continues, it is critical to continue to identify molecular targets and their potential for chemotherapeutic disruption.

Proteases have been under scrutiny as targets of immunological or chemotherapeutic anti-*Schistosoma *agents because of their vital role in many stages of the parasitic life cycle [7,8]. In addition, proteases are known to act as important regulatory elements in a variety of species [9,10]. They also play a vital role as effectors of virulence in pathogens in general, often serving to alter host signal transduction and modify the immune response [11-14]. By targeting proteases specific to parasitic life style or those with significant dissimilarity to homologous proteases in the host species, investigators hope to find anti-schistosomal chemotherapies with minimal side effects to the host. However, few proteolytic enzymes have been purified in *Schistosoma*, and even fewer have well characterized functions and interactions.

Proteases of all five catalytic classes have been identified from *Schistosoma *species through proteomic or genetic analysis. Function has been established for only a handful of identified *S. mansoni *proteases, and the vast majority of these are the digestive proteases involved in metabolic food processing or host tissue penetration [15-18]. Additional proteases that are involved in reproduction, evasion of host immune system, and development have also been characterized [7,19]. Very few proteases have been evaluated for the potential to serve as chemotherapeutic targets against schistosomiasis (e.g. [20]). Fortunately, it is almost certain that additional proteases exist in the *S. mansoni *genome, as the conserved classes of many regulatory proteases have not been identified from *Schistosoma *species. Since current therapies for a wide variety of disorders and diseases target regulatory molecules, such proteases may serve as new and effective targets for anti-helminthic treatments. The challenge of developing new therapies involves several steps, the first of which is to identify and characterize potential targets of drug or vaccine treatments. This is currently a task that is increasingly accomplished and streamlined with genomic and bioinformatic tools [9,21].

The sequencing and annotation of the *S. mansoni *genome [22], combined with large EST libraries, provide a wealth of data from which to identify new vaccine or therapy targets [23-25]. These data, combined with bioinformatics tools and specialized databases, can fast-track the identification of potential anti-trematode agents by complementing and supplementing traditional genetic and proteomic identification techniques. Therefore, as an initial step in characterizing some of these potential targets, we survey the *S. mansoni *genome and EST library for protease genes. In doing so, we identify for the first time, numerous potentially important proteases in trematodes, many of which will have essential functions and may serve as targets of effective chemotherapeutic or immunological treatments.

## Results and Discussion

After culling the data of redundant sequences, inactive homologs and likely pseudogenes, we identified a total of 255 predicted proteases among 54 families (Table 1; see Additional file 1). The 255 proteases identified here comprise 2.1% of the roughly 12,000 predicted genes in the *S. mansoni *genome [22]. In the MEROPS database, there are currently 22 known or putative proteases identified in *S. mansoni*. Since over 90% of the genes presented here are new, this bioinformatic data mining represents a substantial expansion of putative proteases from this species. As expected, proteases of five catalytic classes were identified: 4% are aspartic, 26% are cysteine, 39% are metalloproteases, 24% are serine, and 6% are threonine proteases. These proportions are largely in harmony with those from other organisms [26,27].

**Table 1:** Summary of characteristics from putative protease sequences from the *Schistosoma mansoni *genome.

**Protease class**	**Num. protease sequences**	**Num. protease families**	**Proteases with predicted transmembrane helices**		**Proteases with signal sequence**
			TMHMM	TUPS	
Aspartic	11	3	3	5	4
Cysteine	68	14	1	29	13
Metallo	100	19	19	46	11
Serine	60	15	12	34	16
Threonine	16	3	1	6	1
Totals	255	54	36	120	45

The recently published genome sequence of *S. mansoni *estimates the presence of 335 protease sequences in 60 families [22]. For the protease families represented in both publications, estimated numbers are identical in 19 families, and 27 families are estimated by Berriman et al. [22] to have more members. In cases where estimates differ, most are very similar, the exception being families A01, C01, and S09, which we estimate to have a little more than half the previously published numbers. Such differences are easily attributed to differences in data mining methodologies. For instance, Berriman et al. used a Markov method to identify divergent homologues and culled sequences less than 80 residues or those that overlapped less than 50% of the most similar homologue. In comparison, our methods utilized no specialized methods to identify divergent homologues and culled sequences less than 100 residues or that overlapped less than 80% of the most similar homologue.

**Figure 1:**
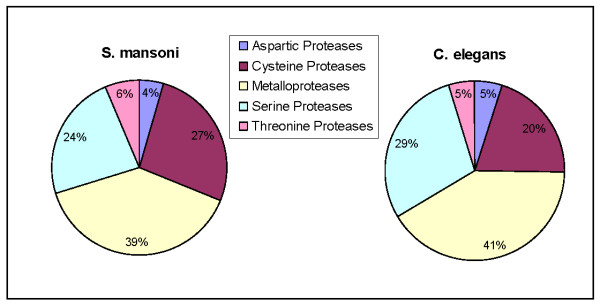
**Comparative graphic showing the relative proportions of each protease class in the species *S. mansoni *and *C. elegans***.

Comparatively, *Caenorhabditis elegans*, a round worm with a well characterized and annotated genome, has 341 known or putative proteases in the MEROPS database. The proportions of classes of proteases are roughly equivalent, but we note a slight expansion in the relative proportion of serine proteases in *C. elegans *compared to *S. mansoni*, and a comparative expansion of cysteine proteases in *S. mansoni *(Figure 1). Some differences will also naturally arise because of differences in organism complexity and life style (*C. elegans *is a free living species, whereas *S. mansoni *is parasitic). However, differences between these two species may also partially be attributable to the coverage and sequence quality of the genomes, and the fact that *S. mansoni *genome characterizations are newer and still very much subject to verification and basic delineation.

**Figure 2:**
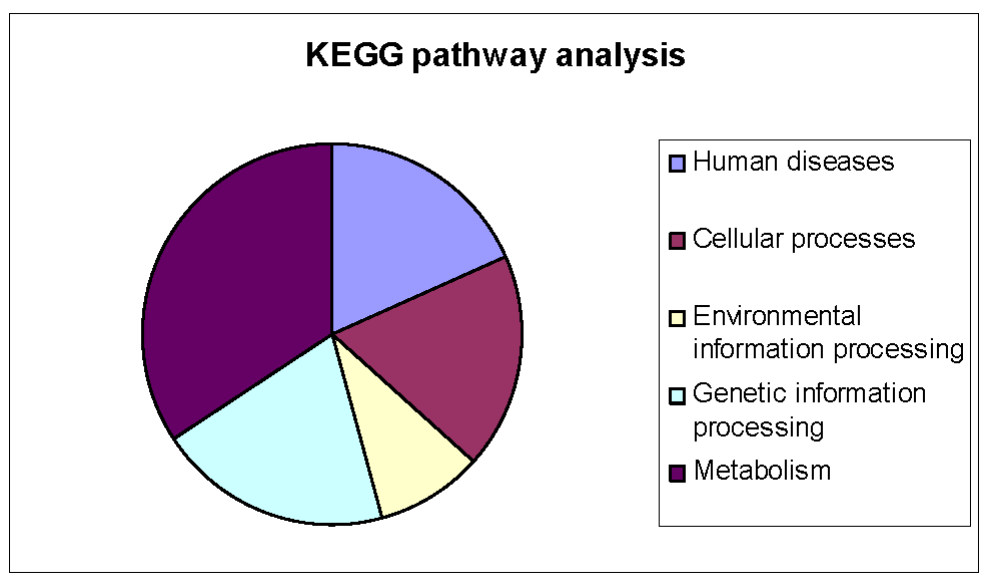
**Results of KEGG analysis on S. mansoni proteases**. The graphic shows the relative proportions of proteases that are involved in various different cellular and metabolic processes and pathways. See Additional file 2 for more details.

Most of the protein sequences that had high similarity to MEROPS database sequences were verified as having a conserved protease-specific domain (see Additional file 1). KAAS was able to assign orthology and KEGG functional pathways to 120 of the *S. mansoni *proteases (Figure 2; see Additional file 2 for full annotation of KEGG pathways). Forty-one proteases are assigned to be involved in metabolic processes, while twenty-four are involved in genetic information processing. Environmental information processes involve eleven proteases, while eleven additional proteases are engaged in cellular processes such as communication and cell cycling. We note that despite the removal of sequences that show similarity to inactive proteases in MEROPS, and assignment of sequences to functional pathways, none of the new proteases identified in the present work have been experimentally shown to be catalytically active.

Below, we review the findings and highlight several important regulatory proteases which are identified for the first time in this species. We focus on regulatory proteases because numerous digestive proteases are already known and being studied in *S. mansoni*, but relatively little is known about the regulatory proteases despite their potential as targets for novel inhibitive chemotherapies.

### Aspartic Proteases

Eleven loci encoding aspartic proteases from just three families were identified. Four of the aspartic proteases, all in the A1 family, were found to contain a signal peptide. Transmembrane (TM) predictions differed between methods: TMHMM found three proteases with a TM domain, all in family A22; TUPS detected a total of 5 TM domains among the A1 and A22 families (Table 1; see Additional file information 1). Two loci were significantly similar to family A2 proteases, but were subsequently found to lack similarity to any conserved domain, protease or not, in the CD database. We found five loci encoding proteins with high similarity to Pepsin/Cathepsin type aspartic proteases, which are broadly distributed among taxa and are most active at an acidic pH. Many of these sequences have already been identified in *S. mansoni*, and are known to be involved in digestion of host proteins [28]. Other functions of this class of proteases are also known, and it is possible that specific sub-functions may be portioned among loci. However, only two of these loci show similarity to sequences in the *S. mansoni *EST library, so this possibility should be investigated further.

We also identify three A22 protease family members from *S. mansoni *for the first time. This class of proteases serves as important regulatory elements through their exceptional ability to lyse peptide bonds within a cell membrane [29]. The capacity to lyse peptide bonds that lie embedded within a plasma membrane (also termed regulated intramembrane proteolysis, or RIP) is shared only among A22, M50 and S54 family proteases [30]. These proteases associate with latent, membrane bound cell signaling molecules; upon intramembrane proteolysis, bioactive domains of the signaling molecules are freed to initiate a signaling cascade or act as an enzyme [31].

**Figure 3:**
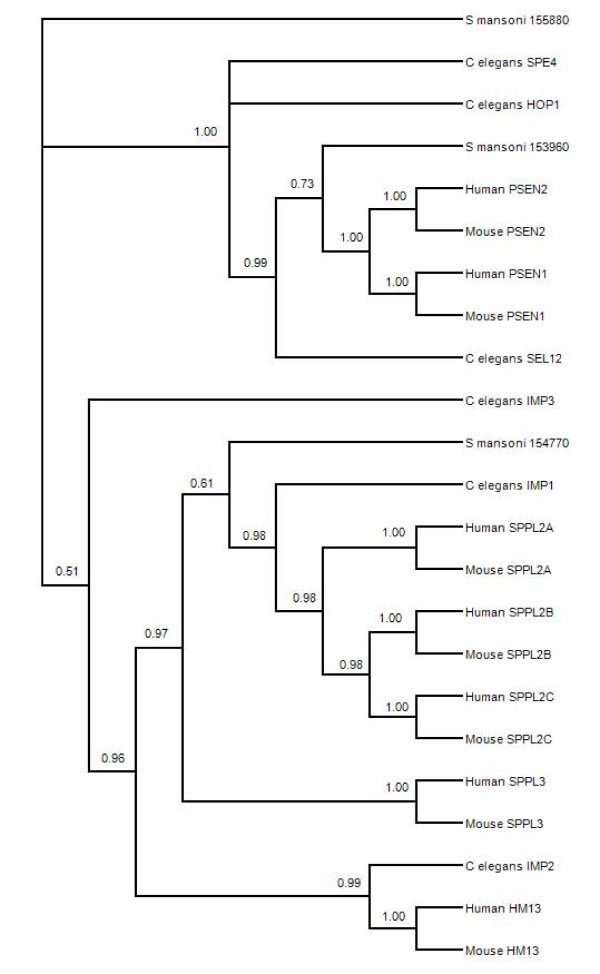
**Consensus phylogenetic tree based on amino acid sequences of selected A22 family proteases**. The topology is inferred using Bayesian criteria, and the consensus topology is obtained by retaining bifurcations with > 50% Bayesian support values. *S. mansoni *sequences are partitioned among nodes known to have functionally diverged in other species.

The *S. mansoni *A22 protease Smp_153960 contains both active site motifs, and in a phylogeny (Figure 3) is closely associated with presenilin-type proteases from *C. elegans*. The conserved evolutionary function of presenilin proteases is the control of NOTCH proteins, which act as regulators of gene expression and are involved in diverse cellular and tissue functions such as cell maturation, angiogenesis, and neuronal function and development [32]. Smp_154770 also contains both active site motifs, but forms part of a clade comprised of signal peptide proteases. The signal peptide proteases (also known as impas-like proteases) are not associated with NOTCH signaling in *C. elegans*, but are involved in embryonic development and larval molting [33]. Impas protease knockout *C. elegans *have an embryonic lethal phenotype, possibly due to the function of the protease in lipid homeostasis and the production of cholesterol-derived hormones. Thus it is significant that Smp_154770 clusters with other signal peptide proteases, which are nonredundant and distinct from presenilin protease activity. The remaining A22 protease from *S. mansoni*, Smp_155880, does not contain the complete catalytic dyad (Additional file 2). Smp_155880 is not allied closely in the phylogenetic tree with either presenilin or signal peptide proteases (Figure 3) but the genetic distance shows it shares higher sequence similarity to Smp_153960 (genetic distance values: Smp_155880/Smp_153960, 0.918; Smp155880/Smp_154770, 0.889; Smp_154770/Smp_153960, 0.871). Interestingly, A22 family proteases cleave the signal peptides of MHC class I molecules (HLA-A, -B, -C) in humans, which serve as a ligand for HLA-E molecules. This activity reports successful MHC biosynthesis to the immune system and regulates NK cell mediated attack [34].

### Cysteine Proteases

We detected 68 loci with significant similarity to known cysteine proteases; of these, 19% had an identifiable signal sequence and thus are likely to enter the secretory pathway. There was a large discrepancy between the two TM domain prediction algorithms: TMHMM found a TM domain in just one cysteine protease, whereas TUPs detected such a domain in 60% of the members of this catalytic class (Table 1). We detected 15 members of the C1 family of proteases, which contain the well-known cysteine-type cathepsin activity involved in digestion of host proteins [18]. Eight of these loci had a conserved cathepsin B domain and other cathepsin family members were detected as well. Cathepsin proteases have already shown promise as targets of new anti-schistosomal therapies and further investigation is warranted [35]. In addition to the digestive enzymes characterized by the cathepsins, we also detected numerous regulatory cysteine proteases.

Regulatory cysteine proteases include the calcium-dependent proteases typified by the calpain and caspase proteases. Calpain proteases belong to the C2 family, of which we found eight members encoded in the *S. mansoni *genome. Calpains are regulatory proteases with a broad array of functions and are involved in cell mobility, cell cycle progression, and the regulation of clotting factors. Calpains are one class of proteases already under investigation as vaccine candidates in *S. japonicum*, with results indicating a reduction in worm burden and egg production in immunized mice [20].

Caspase proteases are also calcium-dependent, and are most prominently known to control apoptosis in a wide variety of animals, including other bilaterian worms [36]. However, the so called inflammatory caspases mediate non-death functions such as inflammation, immunity, and maturation of a variety of cell types [37]. Although there is broad overlap, apoptosis caspases are often categorized as initiator (which are upstream in the signaling cascade and activate other signaling proteins), and effector caspases (which directly lyse cellular proteins whose damage leads to classical signs of apoptosis). The four *S. mansoni *caspases identified here all have the conserved catalytic motifs of His-Gly and Ala-Cys, and are preceded by blocks of hydrophobic residues (Additional file 3). *C. elegans *also has 4 caspase loci, but only three have a conserved catalytic motif. In a sequence identity matrix of *S. mansoni*, *C. elegans *and human caspases, all of the *S. mansoni *caspases shared more similarity to caspases from humans rather than to any other species, including other *S. mansoni *caspases (Table 2). In three out of the four cases, *S. mansoni *shared their highest similarity to human caspases, whereas all human caspases except one were most similar to another human caspase.

**Table 2:** Identity matrix of selected caspase proteases

**Seq->**	**1**	**2**	**3**	**4**	**5**	**6**	**7**	**8**	**9**
1 Smp_032000	ID	0.111	0.187	0.192	0.210	0.188	0.203	**0.220**	0.181
2 Smp_141270	0.111	ID	0.103	0.107	0.095	0.103	**0.133**	0.109	0.102
3 Smp_172010	0.187	0.103	ID	0.286	0.233	**0.314**	0.245	0.221	0.228
4 Smp_028500	0.192	0.107	0.286	ID	0.246	**0.327**	0.244	0.242	0.232
5 Cele_CED3	0.210	0.095	0.233	0.246	ID	0.296	0.267	0.258	0.222
6 Hsap_casp3	0.188	0.103	0.314	**0.327**	0.296	ID	0.319	0.289	0.252
7 Hsap_casp8	0.203	0.133	0.245	0.244	0.267	0.319	ID	0.272	**0.337**
8 Hsap_casp9	0.220	0.109	0.221	0.242	0.258	**0.289**	0.272	ID	0.219
9 Hsap_casp10	0.181	0.102	0.228	0.232	0.222	0.252	**0.337**	0.219	ID

**Figure 4:**
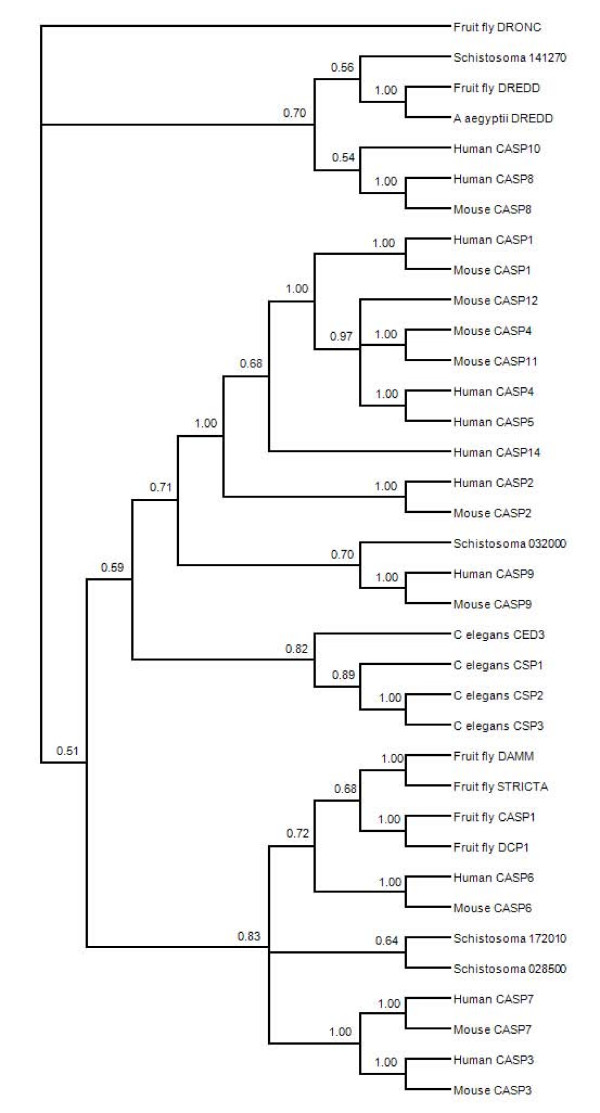
**Consensus phylogenetic tree based on amino acid sequences of selected C14 family (caspase) proteases**. The topology is inferred using Bayesian criteria, and the consensus topology is obtained by retaining bifurcations with > 50% Bayesian support values. Multiple S. mansoni caspase sequences are represented, and these are partitioned among caspases sequences that have undergone functional divergence in other species.

Caspases fall into three phylogenetic clades, which largely correspond with tetrapeptide substrate specificity [38]. Caspases from a species typically fall into multiple clades, with the known exception being *C. elegans*, whose caspases also form a species-specific clade in our phylogenetic analysis. *S. mansoni *caspases are dispersed among the three clades (Figure 4), as seen in most species, but unlike *C. elegans*. Two *S. mansoni *caspases (Smp_172010 and Smp_028500) are in a clade with effector caspases from humans and mice, while Smp_141270 is associated with classical initiators caspase 8 and caspase 10. The fourth trematode caspase is in a monophyletic group with the initiator caspase 9, and a larger clade of mostly inflammatory caspases, which have a regulatory role in the immune system and are associated only with species having a complex hematopoesis involved in the immune response. The placement of *S. mansoni *caspases in the larger phylogeny raises interesting possibilities regarding the specificity and function of these proteases which warrants further investigation. Although caspases are important regulatory proteins and are targets of chemotherapeutic agents in several pathologies, including cancer [39], no anti-schistosomal agents are currently being investigated that target these proteases.

Another group of important regulatory elements include the cysteine proteases that interact with ubiquitin, SUMO and NEDD molecules (hereafter collectively referred to as ub-like). Ub-like molecules are known to function in the targeting of proteins for destruction by the 26S proteasome, but they also play a prominent regulatory role in the cell through post-translational modification, and protein turn-over and half life [40,41]. We have putatively identified more than 30 members from the C12, C19, and C48 proteases families, which interact with ub-like protein molecules. These proteases are responsible for the activation (through cleavage of C-terminal di-glycine motifs) and recycling (by removing ub-like molecules from targeted proteins) of ubiquitin and SUMO molecules, controlling the regulatory roles these molecules play [42]. For instance, ubiquitin is known in part to regulate apoptosis by antagonizing the reaction of caspases with the apoptosis complex; cleavage by ubiquitin proteases is central to this process [43].

It is interesting to note that some of the molecules that effect pathogenesis in bacteria use cysteine proteases that operate on host ub-like molecules to interfere with the regulation of proteins during infection [14]. In fact, numerous effector proteins causing virulence in plant and animal pathogens such as *Yersia *and *Pseudomonas *are cysteine proteases, which target SUMO and other regulatory and signaling molecules such as NF-κB [44].

### Metalloproteases

Metalloproteases make up a large fraction of proteolytic enzymes in the *S. mansoni *genome; one hundred loci were detected, and 12% of these contained signal sequence. Nineteen percent of metalloproteases have one or more trans-membrane alpha helices, indicating that a substantial portion of these proteases may be membrane bound (Table 1). The bioinformatic approach found a locus that may correspond to the previously identified LAP protease of family M17 [45]. Additionally, we found a putative locus for a previously isolated protein thought to be the SmDPPIII, in family M28 [46]. Many other metalloproteases, previously unidentified, were also detected.

There are hundreds of proteins that operate in the mitochondria, and the vast majority of them are encoded in the nucleus. The mitochondrial processing protease (MPP) and the mitochondrial intermediate protease (MIP) often function in concert to cleave signal peptides from immature mitochondrial proteins that are synthesized in the cytoplasm [47]. Failure to do so obstructs further protein sorting, assembly, and function. We detected both the α- and β- (which should be catalytically active) subunits of the *S. mansoni *MPP. The β-MPP of *S. mansoni *has the conserved HxxEHx_(76)_E motif, indicating that it is probably catalytically active (Additional file 3). The *S. mansoni *α-MPP and the β-MPP share 20% identity, which is within the normal range of this comparison for several taxa. Interestingly, *S. mansoni *β-MPP shares 50% sequence identity with its human homolog, which is a higher similarity than the *S. mansoni *- *C. elegans *β-MPP homologs (40%).

A predicted protein for MIP was also detected, and contains a HExxH zinc binding domain, critical to catalysis. The putative *S. mansoni *MIP sequence contains 18 cysteine residues, consistent with cysteine enrichment in the MIP proteins of other species (human and rat MIPs have 18 and 16 cysteine residues respectively) [47]. However, the C terminus of the protein truncates part of the larger catalytic domain conserved in this family of proteases, indicating that this sequence may either be artificially abbreviated by the gene prediction algorithms, or represent a pseudogene.

ATP-dependent mitochondrial proteases are also known, but their function is less well defined. Generally however, these play an essential role in quality control, turnover, and assembly of the respiratory chain complex proteins [48]. Nine ATP-dependent proteases of the M41 family are detected in *S. mansoni*, and all contain an ATP binding motif, but only one sequence (Smp_165550) contains the active site HExxH motif. Treatment of *S. mansoni *with metalloprotease inhibitors results in unexplained paralysis of adult worms [49]. This could be related to mitochondrial ATP-dependent protease inhibition, as defects or loss of this protease results in spastic paraplegia in humans and mice [50].

M50 family proteases are one of the three families of proteases that can perform RIP, and include mammalian S2P proteases, and bacterial SpoIVFB. One protease of the M50 family with an active site motif (HExxH) was detected in our analysis (Smp_054310). This protease is predicted to have multiple transmembrane helices, and the putative active site is found within the third transmembrane helix (approximately residues 175-195; see Additional file 3), consistent with predicted function [31]. As with other proteases that perform RIP, bioactivation of the substrate is a multi-step process, involving multiple proteases. In mammals, S2P cannot perform RIP unless the substrate protein is first cleaved by S1P, a S08 family protease. A homolog of mammalian S1P is also found in *S. mansoni*, providing evidence of an active and at least partially conserved functional mechanism [51].

### Serine Proteases

Sixty serine proteases were detected in our screening of the *S. mansoni *predicted proteins, and these are divided among 15 families. Sixteen loci were found to have a predicted signal sequence--many of these are in the S1 family, which is consistent with the majority of known S1 family proteases entering the secretory pathway and having a signal sequence. Twelve of the serine proteases have at least one transmembrane alpha helix, as predicted by TMHMM, whereas TUPs identified 35 proteases with a TM alpha helix (Table 1). We identified a locus for the well known cercarial elastase [52], and note that several loci also identified as having the highest similarity to cercarial elastase were detected, but were excluded from the analysis due to the likelihood of being pseudogenes. Numerous regulatory serine proteases are identified for the first time in *S. mansoni *with this report.

**Figure 5:**
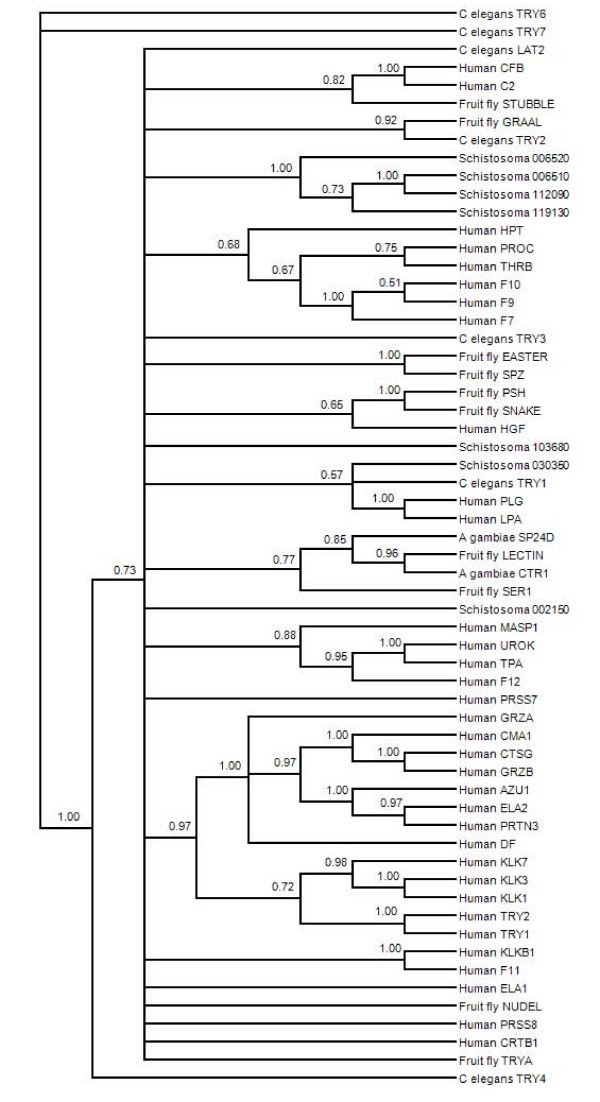
**Consensus phylogenetic tree based on amino acid sequences of selected S1 family (chymotrypsin) proteases**. The topology is inferred using Bayesian criteria, and the consensus topology is obtained by retaining bifurcations with > 50% Bayesian support values. Multiple *S. mansoni *proteases are represented, many of which form a tight cluster with other *S. mansoni *S1 proteases and are divergent from functionally defined S1 proteases of other species.

The S01 family is the largest group of proteases, with a wide variety of functions. This functional divergence is frequently reflected in phylogenetic grouping and/or variation at 5-6 base-pair motifs surrounding functionally important residues, such as active sites [53-55]. We identified eighteen S01 proteases in the *S. mansoni *genome, however six of these did not have a significant similarity to a conserved protease domain (Appendix 1). In addition, alignment of the sequences revealed that only seven of these predicted proteins have the conserved catalytic triad of His, Asp, and Ser (Additional file 3). A phylogeny of S01 proteases from human, *Drosophila*, *C. elegans*, and *S. mansoni *resulted in a general lack of resolution in the deeper nodes, making it difficult to ascertain any level of functional clustering (Figure 5). Despite this, a strongly supported cluster of four *S. mansoni *proteases (Smp_06510, _06520, _112090, and _119130) is formed among sequences with 58-99% identity. Included in this cluster are cercarial elastase and other proteases of similar predicted length. Two of the remaining *S. mansoni *S01 proteases are in a large unresolved polytomy that includes S01 proteases from all species included in the analysis. The final *S. mansoni *S01 protease is in a clade of "effector" proteases that function downstream in a signaling cascade and operate on cellular substrates (such as elastase and plasminogen). Although it is impossible to assign functions to *S. mansoni *S01 proteases through the above analysis, results indicate a possible functional divergence among *S. mansoni *S01 proteases. Some of these sequences may have a closely related (original) function that is not shared with other organisms included in the analysis.

We also found members of the S08 family that have a catalytic mechanism that is distinct from the typical chymotrypsin activity of S01 family proteases. This family is mostly comprised of endoproteases with broad function, many of which enter the secretory pathway [56]. *S. mansoni *also has S26 family members, which contain proteases that are responsible for processing numerous precursor proteins to active, mature forms [57]. This occurs when signal peptides of newly synthesized proteins are cleaved upon arrival at a functional site. This cleavage event also acts as a post-translational regulatory event, controlling the activation of these proteins until the signal peptide is removed. Other AAA mitochondrial proteases of the S16 family, which bind DNA and RNA and may participate directly in the metabolism of mtDNA, are also found. Downregulation of this protease causes a general activation of caspases and leads to apoptosis [58].

We also found members of the S54 family of proteases in the *S. mansoni *genome. This family of proteases functions to perform RIP, resulting in the liberation of bioactive signaling peptides from anchoring TM domains [13,59]. Consistent with the expected function, these S54 proteases have several TM helices predicted by both TMHMM and TUPs, indicating a likely conserved structure among taxa (Appendix 1).

**Figure 6:**
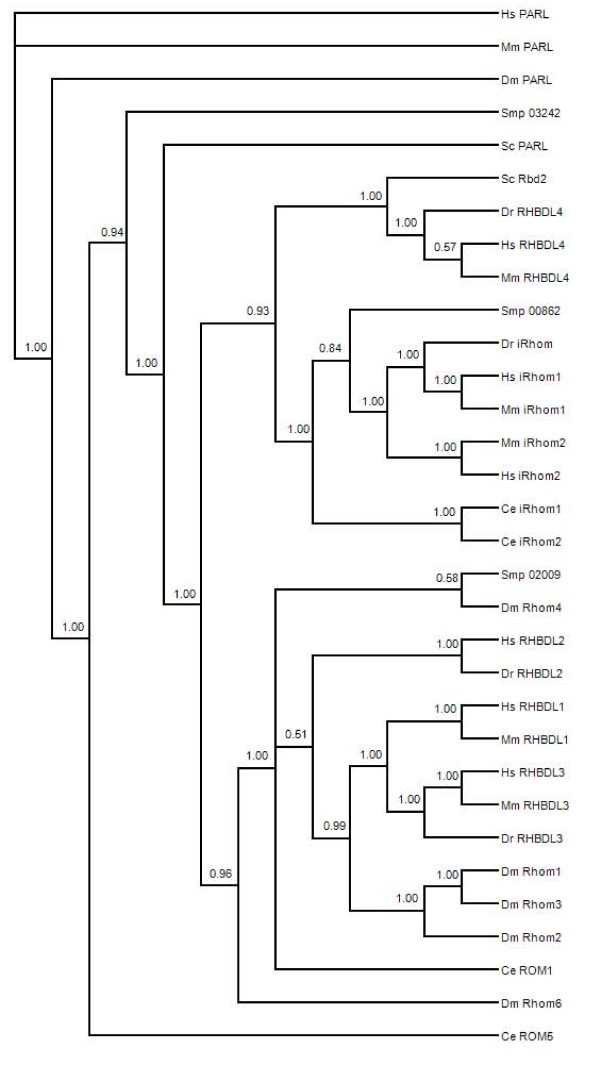
**Consensus phylogenetic tree based on amino acid sequences of selected S54 family (rhomboid) proteases**. The topology is inferred using Bayesian criteria, and the consensus topology is obtained by retaining bifurcations with > 50%. Bayesian support values. Multiple *S. mansoni *rhomboid proteases are represented and these are partitioned among various classes of functionally divergent rhomboids from other species.

S54 family proteases are termed rhomboid proteases and can be divided into several groups because of conserved structure, substrate similarity, and cellular localization. These differences are reflected in their phylogenetic clustering into PARL, secretase, and iRhom-type proteases [60]. We included putative *S. mansoni *rhomboid proteases in phylogenetic analysis to categorize these into subtypes and as an initial assessment of similarity and possible function (Figure 6). The resulting phylogenetic tree strongly supported groups corresponding to PARL, secretase, and iRhom proteases, but the relationships within and among the three groups were not always clear. The three *S. mansoni *putative rhomboid family members fell into three separate clades. Smp_032420 has the conserved histidine and GxSx motif in a hydrophobic region predicted to be in a transmembrane helix (see Additional file 3). This is characteristic of proteolytically active rhomboids and Smp_032420 was placed with the PARL-like proteases, which localize to the mitochondria. Smp_020090 clusters with the secretase-A rhomboids, and consistent with this clustering is the conserved catalytic motif in a hydrophobic region. Smp_008620 is short and likely represents an incomplete sequence, but portions of it do show significant similarity to iRhom-like rhomboids, including a conserved proline in the degenerate catalytic motif. iRhoms lack a catalytic activity, but their phylogenetic clustering suggests some conserved functional relationship. These assessments of *S. mansoni *rhomboids to functional classes are simply based on similarity and await experimental confirmation.

### Threonine Proteases

Threonine proteases are most closely associated with the elements of the 20S proteasome, and the majority of threonine proteases discovered in our analysis appear to be subunits of that proteasome. At least 5 alpha subunits and 4 beta subunits of the proteasome were detected, all of which show evidence of being expressed. *S. mansoni *proteasome elements have already been identified and characterized through proteomic analysis, which detected all seven alpha and beta subunits and displayed morphological diversity due mainly to post-translational modifications [61]. Thus, the bioinformatic data mining done here failed to identify all threonine protease genes present in the genome, or the genome annotation and gene detection methods used have failed to detect some genes known to be present. This difference highlights the need for collaborative work and the complementary nature of bioinformatic, and proteomic discovery of genetic elements.

In addition to the proteasome subunits, a taspase-like protease was identified in the *S. mansoni *genome. This protease cleaves the general transcription factor TFIIA, regulating transcription of numerous gene products.

## Conclusion

We used *in silico *methods to explore the catalog of predicted proteins of *S. mansoni *for sequences homologous to known proteases. The search resulted in the detection of 255 putative proteases, over 90% of which are recognized for the first time in this species. Along with the proteases already described as having a function primarily in digestion and host invasion, we identify numerous previously unidentified regulatory proteases involved in a variety of biological processes. We focused on regulatory proteases because they generally serve essential functions in the maintenance of homeostasis and the developmental progression of the life cycle of a species. The vital nature of protease-mediated regulatory function underscores the potential of these proteases as targets of inhibitory chemotherapies and we hope that the identification of proteases stimulates further research into this area, such as expanding the application of gene silencing to identify the function of various proteases in *Schistosoma *species [62,63].

## Methods

We used the complete set of protease core sequences from the MEROPS (release 7.8) database [64] to identify putative homologues of known proteases in the *S. mansoni *genome. Core protease sequences comprise a non-redundant library of the catalytic unit of a protease and exclude all other functional units, such as ATP-binding, or calcium-binding domains. These sequences were used to avoid false positive identification of proteins as proteases due to high homology in non-catalytic parts of the sequence. Core sequences were compared to predicted proteins from the annotated *S. mansoni *genome. Release 4.0 of the *S. mansoni *genome includes a first-pass annotation and gene prediction, of which we downloaded the complete database of predicted proteins. In addition, the complete set of ESTs (release 6.0 of the *S. mansoni *gene index) isolated from 6 life stages of *S. mansoni *was also acquired.

Using the MEROPS proteases as the database, we used the predicted proteins as the query sequences in a local BLASTP [65] comparison. Default parameters were used during the search, where all predicted proteins were queried against all members of the protease database. In the comparison, only sequences with similarity scores <1e-04 were retained as *S. mansoni *protease homologs. From the initial result, query sequences identified as similar to homologous non-protease sequences (protease-like sequences but with mutations in active sites) were culled from the results, as were alternative splice forms of a gene. Additionally, predicted proteins that were shorter than 100 residues or less than 80% of the protease core sequence were not retained. Remaining sequences were subject to full analysis.

Results from the BLASTP query were subject to a number of analyses to characterize the sequences. We first independently sought the predicted function of *S. mansoni *sequences by searching for conserved motif and domains in the protein sequences. This was done using searches in the Conserved Domain Database (CDD) v. 2.13 of NCBI [66,67]. CDD searches employ a reverse position-specific BLAST to align query sequence to protein domains from SMART v. 5.0 [68], Pfam v. 22.0 [69], and COG [70]. The KEGG automated annotation server (KAAS) was used to assign pathway-based functional orthology to sequences in our dataset [71]. We were also interested in identifying alpha-helix domains that likely span a cellular membrane. The prediction of these transmembrane helices is imprecise, so we report the results of two methods: TMHMM [72] and TUP [73].

The expected cellular location (e.g. cytoplasmic, membrane, or mitochondrial) and potential to enter the secretory pathway of a cell is also informative in classifying newly identified proteins. Therefore, we identified signal sequences in the proteins with signalP, using both the neural network and HMM methods [74,75]. The D score is the average of the maximal Y-score (the most likely location of the cleavage site of the signal sequence) and the mean S-score, and is the best way to discriminate true signal sequences in proteins [76]. Proteins with a D score greater than 55 and HMM greater than 90% were scored as having an N-terminal signal sequence and the Y-score was recorded for these proteins. We used the eukaryote setting with each sequence truncated after 70 residues to avoid false positive detection of signal sequence outside of the N-terminus.

Functionally distinct proteases may show differential phylogenetic clustering. Therefore, we performed phylogenetic comparisons, including mainly species with functionally defined proteases with *S. mansoni *sequences (accession numbers of sequences used are given in the Additional files). While phylogenetic analysis does detect evidence of divergence, the functional implications of the divergence must be tested experimentally, as such phylogenetic analyses represent an initial assessment of potential functional divergence among multiple *S. mansoni *proteases within a family. For phylogenetic analysis, homologous protein sequences from selected species were obtained from NCBI GENBANK. Alignments were made using MUSCLE default parameters [77]. Resulting alignments were subject to phylogenetic analysis using MRBAYES [78]. Flexible priors were used by employing a mixed model analysis. Mixed model analysis avoids reliance on a single model of amino acid substitutions (and thus a single prior) by allowing the MCMC sampler to regularly propose and explore the fit of new models during analysis (see MRBAYES documentation for model specifications). Each model contributes to the results in proportion to their posterior probability. We performed two simultaneous but independent runs on each data set, with each run consisting of three heated chains and one cold chain. Runs were continued until the average standard deviation of the split frequencies between the two runs was less than 0.02, with a minimum of 100,000 MCMC generations. Trees were sampled every 50^th ^generation, resulting in at least 2,000 saved trees. The first 25% of trees were discarded as burn-in prior to summarizing sampled trees. Summarizing samples produced a consensus tree with branch bifurcation support (clade credibility) indicated. Clade credibility was calculated for each bifurcation as the proportion of sampled trees with that bifurcation.

## Authors' contributions

DHB conceived of the study, participated in study design, carried out analysis, and drafted the manuscript. CM participated in study design, wrote computer code to automate analysis, and helped draft and revise the manuscript. DJM participated in study design, and helped draft and revise the manuscript. All authors read and approved the final manuscript.

## Supplementary Material

Additional file 1 **Tables of *S. mansoni *sequences that have significant similarity to known proteases**. Tables of results showing which S. mansoni sequences have significant similarity to known proteases, which protease family they belong to, protease conserved domains present, and presence of a signal sequence or transmembrane regions. Click here for file

Additional file 2 **KAAS analysis: KEGG pathway assignment and KEGG orthology number (KO number) of each *S. mansoni *protease**. Bioinformatic analysis using the Kyoto Encyclopedia of Genes and Genomes used to predict probable functions and the cellular processes for *S. mansoni *proteases, based on orthologous relationships of proteases for which function is clearly established in other species. Click here for file

Additional file 3 **Figures of catalytic motifs and active sites of selected protease families**. A series of six figures, each of which shows a partial sequence alignment of a family of proteases for several species. Highlighted are catalytic motifs which are directly involved in catalysis in that family of proteases, and the active site itself, which is essential to the proteolytic function. Click here for file
