# Supplementary material for: Analysis of regulatory protease sequences identified through bioinformatic data mining of the Schistosoma mansoni genome
Source: BMC Genomics. 2009 Oct 21;10:488. doi: 10.1186/1471-2164-10-488 (PMC2772863; doi:10.1186/1471-2164-10-488)
Supplement: Additional file 1 — Tables of S. mansoni sequences that have significant similarity to known proteases. Tables of results showing which S. mansoni sequences have significant similarity to known proteases, which protease family they belong to, protease conserved domains present, and presence of a signal sequence or transmembrane regions. [file 1471-2164-10-488-S1.DOC]

|  |  | Aspartic proteases |  |  |  |  |  |
| --- | --- | --- | --- | --- | --- | --- | --- |
| Family | Protease | domain | CDD | eval | signalP | tmhmm | tup |
| A1 | Smp_132480 | Aspartic peptidase, pepsin/cathepsin | Pfam00026 | 3e-66 | 16 |  |  |
|  | Smp_136830 | Aspartic peptidase, pepsin/cathepsin | Pfam00026 | 6e-54 | 16 |  |  |
|  | Smp_013040 | Aspartic peptidase, pepsin/cathepsin | Pfam00026 | 2e-103 | 14 |  | 2 |
|  | Smp_136730 | Aspartic peptidase, pepsin/cathepsin | Pfam00026 | 7e-92 | 18 |  |  |
|  | Smp_175560 | Aspartic peptidase, pepsin/cathepsin | Pfam00026 | 3e-05 |  |  |  |
| A2A | Smp_121030 | Retroviral aspartyl protease | Pfam00077 | 1e-14 |  |  |  |
| A2X | Smp_190380 | None | na | na |  |  | 1 |
|  | Smp_154310 | None | na | na |  |  |  |
| A22 | Smp_153960 | Presenilin | Pfam01080 | 2e-92 |  | 7 | 10 |
|  | Smp_154770 | Signal peptidase | Pfam04258 | 3e-27 |  | 9 | 11 |
|  | Smp_155880 | **Galactosyltransferase** | Pfam02709 | 4e-39 |  | 1 | 1 |
|  |  |  |  |  |  |  |  |
|  |  |  |  |  |  |  |  |
|  |  |  |  |  |  |  |  |
|  |  |  |  |  |  |  |  |
|  |  |  |  |  |  |  |  |

Additional file 1. Tables of *S. mansoni* sequences that have significant similarity to known proteases.

Table S1: Aspartic proteases

Table S2: Cysteine proteases

|  |  | Cysteiene proteases |  |  |  |  |  |
| --- | --- | --- | --- | --- | --- | --- | --- |
| family | ID | conserved domains | domain family | eval | signal | TMHMM | TUP |
| C01 | Smp_157090 | C1A peptidase, papain | cd2248 | 3e-85 | 17 |  | 2 |
|  | Smp_179950 | C1A peptidase, cathepsinB | cd2620 | 1e-78 | 18 |  |  |
|  | Smp_158420 | C1A peptidase, cathepsinB | cd2620 | 2e-97 | 18 |  |  |
|  | Smp_103610 | C1A peptidase, cathepsinB | cd2620 | 5e-93 | 18 |  |  |
|  | Smp_067060 | C1A peptidase, cathepsinB | cd2620 | 2e-93 | 23 |  |  |
|  | Smp_034410 | C1A peptidase, papain | cd2248 | 2e-48 | 17 |  | 2 |
|  | Smp_019030 | C1A peptidase, cathepsinC | cd2621 | 1e-94 | 21 |  | 1 |
|  | Smp_141610 | C1A peptidase, cathepsinB | cd2620 | 1e-92 | 23 |  |  |
|  | Smp_139160 | C1A peptidase, papain | cd2248 | 1e-40 | 18 |  | 1 |
|  | Smp_193000 | C1A peptidase, papain | cd2248 | 8e-35 | 18 |  | 1 |
|  | Smp_149730 | C1A peptidase, papain | cd2248 | 1e-39 |  |  |  |
|  | Smp_085010 | C1A peptidase, cathepsinB | cd2620 | 2e-73 | 21 |  |  |
|  | Smp_105370 | C1A peptidase, cathepsinB | cd2620 | 7e-74 |  |  |  |
|  | Smp_181030 | C1A peptidase, cathepsinB | cd2620 | 7e-74 |  |  | 2 |
|  | Smp_139240 | C1A peptidase, papain | cd2248 | 7e-58 | 21 |  | 1 |
| C02 | Smp_089460 | Calpain, domain II | cd00044 | 5e-97 |  |  |  |
|  | Smp_003980 | Calpain, domain II | cd00044 | 3e-91 |  |  | 2 |
|  | Smp_137410 | Calpain, domain II | cd00044 | 4e-66 |  |  |  |
|  | Smp_159550 | Calpain, domain II | cd00044 | 2e-41 |  |  |  |
|  | Smp_130640 | Calpain, domain II | cd00044 | 2e-06 |  |  | 3 |
|  | Smp_167480 | Calpain, domain II | cd00044 | 1e-65 |  |  | 3 |
|  | Smp_083530 | Calpain, domain II | cd00044 | 1e-65 |  |  | 2 |
|  | Smp_157500 | Calpain, domain II | cd00044 | 1e-96 |  |  | 2 |
| C12 | Smp_147920 | Ubiquitin hydrolase | Pfam01088 | 2e-29 |  |  | 3 |
|  | Smp_083200 | Ubiquitin hydrolase | Pfam01088 | 2e-46 |  |  |  |
|  | Smp_168800 | Ubiquitin hydrolase | Pfam01088 | 5e-36 |  |  | 3 |
| C13 | Smp_011230 | Asparaginyl peptidase; legumain | Pfam01650 | 1e-26 |  |  |  |
|  | Smp_075800 | Asparaginyl peptidase; legumain | Pfam01650 | 1e-101 |  |  |  |
| C14 | Smp_032000 | Caspase | cd00032 | 1e-56 |  |  | 1 |
|  | Smp_028500 | Caspase | cd00032 | 8e-63 |  |  |  |
|  | Smp_141270 | Caspase | cd00032 | 5e-53 |  |  |  |
|  | Smp_172010 | Caspase | cd00032 | 5e-56 |  |  |  |
| C15 | Smp_140810 | Pyroglutamyl peptidase | cd00501 | 2e-18 |  |  |  |
| C19 | Smp_021300 | Ubiquitinyl hydrolase | cd02674 | 1e-23 |  |  | 2 |
|  | Smp_047360 | **UBX Ubiquitin-like domain** | cd01772 | 6e-17 |  |  |  |
|  | Smp_125860 | Ubiquitinyl hydrolase | cd02668 | 1e-42 |  |  |  |
|  | Smp_046430 | Ubiquitinyl hydrolase | cd02661 | 4e-73 |  |  | 2 |
|  | Smp_074200 | Ubiquitinyl hydrolase | cd02661 | 8e-21 |  |  | 2 |
|  | Smp_175520 | Ubiquitinyl hydrolase | cd02674 | 1e-56 |  |  |  |
|  | Smp_000710 | Ubiquitinyl hydrolase | cd02663 | 3e-117 |  |  |  |
|  | Smp_122440 | Ubiquitinyl hydrolase | cd02663 | 3e-118 |  |  |  |
|  | Smp_089180 | Ubiquitinyl hydrolase | cd02659 | 7e-112 |  |  |  |
|  | Smp_074400 | Ubiquitinyl hydrolase | cd02660 | 1e-70 |  |  | 2 |
|  | Smp_123630 | Ubiquitinyl hydrolase | cd02257 | 4e-24 |  |  |  |
|  | Smp_058560 | Ubiquitinyl hydrolase | cd02659 | 1e-21 |  |  |  |
|  | Smp_153690 | Ubiquitinyl hydrolase | cd02659 | 5e-32 |  |  |  |
|  | Smp_017890 | Ubiquitinyl hydrolase | cd02669 | 3e-166 |  |  |  |
|  | Smp_128770 | Ubiquitinyl hydrolase | COG5560 | 8e-100 |  |  |  |
|  | Smp_069960 | Ubiquitinyl hydrolase | COG5207 | 9e-67 |  |  |  |
|  | Smp_152000 | Ubiquitinyl hydrolase | cd02674 | 3e-56 | 23 |  |  |
|  | Smp_162200 | Ubiquitinyl hydrolase | cd02667 | 1e-24 |  |  | 1 |
|  | Smp_131570 | Ubiquitinyl hydrolase | cd02659 | 2e-73 |  |  | 10 |
|  | Smp_168120 | Ubiquitinyl hydrolase | cd02657 | 4e-42 |  |  | 2 |
|  | Smp_038140 | **Chromosome segregation ATPase** | COG1196 | 1e-05 |  |  |  |
|  | Smp_005280 | Ubiquitinyl hydrolase | cd02257 | 3e-20 |  |  |  |
|  | Smp_122960 | Ubiquitinyl hydrolase | cd02667 | 3e-09 |  | 1 | 2 |
| C26 | Smp_063830 | **Glutamine amidotransferase** | cd01742 | 1e-62 |  |  | 1 |
|  | Smp_134650 | **CTP synthase** | cd03113 | 9e-97 |  |  | 3 |
| C44 | Smp_136260 | **Glutamine amidotransferase** | cd00714 | 4e-63 |  |  | 4 |
| C48 | Smp_033260 | Ulp1 protease | Pfam02902 | 7e-33 |  |  |  |
|  | Smp_159120 | Ulp1 protease | COG5160 | 1e-21 |  |  | 2 |
|  | Smp_121810 | Ulp1 protease | Pfam2902 | 1e-05 |  |  | 1 |
| C50 | Smp_167810 | Separase | COG5155 | 2e-28 |  |  | 2 |
| C54 | Smp_039820 | C54 family peptidase | Pfam03416 | 3e-42 |  |  |  |
|  | Smp_124830 | C54 family peptidase | Pfam03416 | 5e-63 |  |  |  |
| C56 | Smp_082030 | Glutamine amidotransferase | cd03135 | 1e-43 |  |  |  |
| C64 | Smp_052350 | Ovarian tumor-like protease | Pfam02338 | 6e-08 |  |  | 2 |
|  |  |  |  |  |  |  |  |
|  |  |  |  |  |  |  |  |
|  |  |  |  |  |  |  |  |

Table S3 Metalloproteases

|  |  | Metalloproteases |  |  |  |  |  |
| --- | --- | --- | --- | --- | --- | --- | --- |
| family | Gene ID | conserved domain | domain family | eval | signal | TMHMM | TUPs |
| M01 | Smp_173030 | M1 aminopeptidase | Pfam01433 | 1e-97 |  |  |  |
|  | Smp_128960 | M1 aminopeptidase | Pfam01433 | 2e-13 |  |  | 5 |
|  | Smp_007550 | M1 aminopeptidase  Leukotriene A4 hydrolase | Pfam01433  Pfam09127 | 2e-65  2e-23 |  |  |  |
|  | Smp_091470 | M1 aminopeptidase | Pfam01433 | 5e-111 |  |  | 2 |
|  | Smp_174530 | M1 aminopeptidase  Trypsin-like serine protease | Pfam01433  cd000190 | 2e-80  4e-41 |  |  |  |
| M03 | Smp_075220 | Zn-dependent oligopeptidase | COG0339 | 5e-69 |  |  |  |
|  | Smp_029500 | Zn-dependent oligopeptidase | COG0339 | 3e-121 |  |  |  |
|  | Smp_029470 | Zn-dependent oligopeptidase | COG0339 | 6e-107 |  |  | 1 |
| M08 | Smp_127030 | Leishmanolysin | Pfam01457 | 1e-49 |  | 1 | 4 |
|  | Smp_171330 | Leishmanolysin | Pfam01457 | 7e-46 |  |  |  |
|  | Smp_090100 | Leishmanolysin | Pfam01457 | 1e-45 | 24 | 1 | 3 |
|  | Smp_153930 | Leishmanolysin | Pfam01457 | 7e-48 |  |  |  |
|  | Smp_135530 | Leishmanolysin | Pfam01457 | 1e-42 |  |  |  |
|  | Smp_173070 | Leishmanolysin | Pfam01457 | 3e-35 | 24 |  | 1 |
|  | Smp_167090 | Leishmanolysin | Pfam01457 | 7e-28 |  |  |  |
|  | Smp_171340 | Leishmanolysin | Pfam01457 | 9e-45 |  |  |  |
|  | Smp_090110 | Leishmanolysin | Pfam01457 | 1e-45 | 24 | 1 | 4 |
|  | Smp_167120 | Leishmanolysin | Pfam01457 | 4e-29 |  |  | 1 |
|  | Smp_167100 | Leishmanolysin | Pfam01457 | 3e-20 |  |  |  |
| M10 | Smp_162300 | Fibronectin type II domain | cd00062 | 5e-07 |  | 1 | 2 |
|  | Smp_145930 | Matrix metalloproteinase | cd04278 | 5e-52 |  |  |  |
| M12 | Smp_134430 | Zn-dependent metalloprotease | cd04281 | 2e-87 |  |  | 3 |
|  | Smp_047460 | Zn-dependent metalloprotease | cd04280 | 1e-34 |  |  |  |
|  | Smp_160620 | ADAM-like metalloprotease | cd04269 | 6e-21 | 23 | 1 | 4 |
|  | Smp_175340 | ADAM-like metalloprotease | cd04270 | 6e-45 |  |  |  |
|  | Smp_171690 | Thrombospondin-like | cd00090 | 4e-06 |  |  |  |
|  | Smp_145900 | ADAM-like metalloprotease | cd04270 | 1e-42 | 24 | 1 | 2 |
|  | Smp_124500 | ADAMTS-like metalloprotease | cd04273 | 2e-22 |  |  |  |
|  | Smp_146730 | ADAMTS-like metalloprotease | cd04273 | 1e-32 | 28 | 1 | 1 |
|  | Smp_163920 | **Immunoglobulin cell adhesion** | cd00931 | 5e-13 |  |  | 4 |
| M13 | Smp_194190 | M13 peptidase | Pfam01431 | 5e-06 |  |  |  |
|  | Smp_122850 | none |  |  |  |  | 4 |
|  | Smp_159370 | M13 peptidase | Pfam05649 | 5e-30 |  |  |  |
|  | Smp_170500 | M13 peptidase | Pfam01431 | 3e-10 |  |  |  |
|  | Smp_170470 | M13 peptidase | Pfam05649 | 8e-05 |  |  |  |
|  | Smp_181710 | none |  |  |  | 1 | 1 |
|  | Smp_157440 | M13 peptidase | Pfam01431 | 1e-24 | 20 |  | 4 |
|  | Smp_122860 | M13 peptidase | Pfam01431 | 6e-07 |  |  | 2 |
|  | Smp_171100 | M13 peptidase | Pfam01431 | 4e-12 |  |  |  |
|  | Smp_157400 | M13 peptidase | Pfam01431 | 7e-21 |  |  |  |
|  | Smp_173160 | M13 peptidase | Pfam01431 | 9e-09 |  |  |  |
|  | Smp_122450 | M13 peptidase | Pfam05649 | 1e-13 |  | 2 | 5 |
|  | Smp_000760 | M13 peptidase | Pfam05649 | 8e-14 |  | 2 | 5 |
| M14 | Smp_088270 | M14 Zn carboxypeptidase | Pfam00246 | 1e-66 |  |  |  |
|  | Smp_159890 | M14 Zn carboxypeptidase | Pfam00246 | 9e-59 | 23 | 1 | 3 |
|  | Smp_167640 | M14 Zn carboxypeptidase | Pfam00246 | 2e-25 |  |  |  |
|  | Smp_181340 | M14 Zn carboxypeptidase | Pfam00246 | 1e-16 |  |  |  |
|  | Smp_125080 | M14 Zn carboxypeptidase | Pfam00246 | 4e-17 |  |  | 3 |
|  | Smp_127610 | M14 Zn carboxypeptidase | Pfam00246 | 2e-14 |  |  | 2 |
|  | Smp_142190 | M14 Zn carboxypeptidase | Pfam00246 | 1e-42 | 16 |  | 1 |
|  | Smp_082250 | M14 Zn carboxypeptidase | Pfam00246 | 2e-21 |  |  |  |
| M16 | Smp_128100 | Insulinase | Pfam00675 | 4e-32 |  |  | 1 |
|  | Smp_061510 | Insulinase | Pfam00675 | 1e-22 |  |  | 3 |
|  | Smp_156960 | Insulinase | Pfam00675 | 2e-14 |  |  |  |
|  | Smp_155230 | Insulinase | Pfam00675 | 6e-12 |  |  | 2 |
|  | Smp_094050 | Insulinase | Pfam00675 | 2e-21 |  |  |  |
|  | Smp_079450 | Insulinase | Pfam00675 | 2e-12 |  |  |  |
|  | Smp_009650 | Insulinase | Pfam00675 | 5e-49 |  |  |  |
|  | Smp_146140 | M16C associated domain | Pfam08367 | 2e-48 |  |  | 2 |
|  | Smp_155220 | periplasmic Zn protease | COG1025 | 2e-38 |  |  |  |
| M17 | Smp_030000 | cytosol aminopeptidase | cd00433 | 7e-72 |  |  | 1 |
|  | Smp_083870 | cytosol aminopeptidase | cd00433 | 1e-55 |  |  | 2 |
|  | Smp_138380 | cytosol aminopeptidase | cd00433 | 2e-79 |  |  | 2 |
|  | Smp_159960 | M18 Zn aminopeptidase | Pfam02127 | 6e-131 |  |  |  |
| M20 | Smp_019630 | M20 glutamine carboxypeptidase | Pfam07687 | 1e-08 |  |  |  |
|  | Smp_111420 | peptidase T | PRK05469 | 1e-170 |  |  |  |
| M22 | Smp_125030 | O-sialoglycoprotein endopeptidase | PRK09604 | 2e-62 |  |  | 2 |
| M24 | Smp_142010 | methionine aminopeptidase | cd01086 | 5e-90 |  | 3 | 4 |
|  | Smp_159490 | methionine aminopeptidase | cd01086 | 2e-62 |  |  | 5 |
|  | Smp_011120 | methionine aminopeptidase | cd01088 | 2e-132 |  |  |  |
|  | Smp_050070 | X-prolyl aminopeptidase | cd01085 | 3e-62 |  |  |  |
|  | Smp_148960 | X-prolyl aminopeptidase | cd01085 | 3e-62 |  |  |  |
|  | Smp_090800 | Xaa-pro dipeptidase | cd01087 | 3e-63 |  |  |  |
|  | Smp_127540 | Xaa-pro dipeptidase | cd01087 | 1e-28 |  |  |  |
|  | Smp_088280 | related to aminopeptidase P | cd01091 | 2e-63 |  |  | 3 |
|  | Smp_150690 | related to aminopeptidase M | cd01089 | 3e-52 |  |  |  |
| M28 | Smp_135510 | glutamate carboxypeptidase II | cd02121 | 1e-23 |  |  | 2 |
|  | Smp_175220 | M28 peptidase | Pfam04389 | 2e-29 |  | 1 | 3 |
|  | Smp_170400 | M28 peptidase | Pfam04389 | 2e-09 | 40 | 2 | 2 |
| M38 | Smp_085110 | dihydropyrimidinase | cd01314 | 3e-145 |  |  |  |
|  | Smp_106150 | dihydroorotase | cd01316 | 5e-115 |  |  | 4 |
|  | Smp_033660 | **N-acetylglucosamine-6-phosphate deactylase** | cd00854 | 1e-81 |  |  |  |
|  | Smp_078780 | dihydropyrimidinase | cd01314 | 4e-79 |  |  |  |
|  | Smp_126390 | dihydropyrimidinase | cd01314 | 3e-19 |  |  |  |
| M41 | Smp_018620 | ATP-dependent Zn protease | COG0465 | 1e-92 |  |  | 1 |
|  | Smp_119310 | ATP-dep. proteasome regulatory unit | COG1222 | 8e-150 |  |  | 1 |
|  | Smp_012470 | ATP-dep. proteasome regulatory unit | COG1222 | 9e-151 |  |  |  |
|  | Smp_018240 | AAA+ class ATPase | COG0464 | 1e-64 |  |  |  |
|  | Smp_173840 | ATP-dep. proteasome regulatory unit | COG1222 | 2e-151 |  |  |  |
|  | Smp_126110 | ATPase | Pfam0004 | 6e-47 |  |  |  |
|  | Smp_165550 | M41 peptidase  FtsH protease | Pfam01434  Pfam06480 | 5e-55  2e-15 |  | 2 | 2 |
|  | Smp_055760 | ATP dependent Zn protease | COG0465 | 6e-105 |  | 1 | 2 |
| M48 | Smp_082620 | M48 peptidase | Pfam01435 | 1e-24 |  | 7 | 7 |
| M49 | Smp_019010 | M49 peptidase | Pfam03571 | 3e-155 |  |  |  |
| M50 | Smp_054310 | M50 peptidase | Pfam02163 | 4e-08 | 16 | 8 | 9 |
|  | Smp_178770 | **PDZ domain** | Pfam00992 | 5e-07 |  |  |  |
| M67 | Smp_126690 | **proteasome regulatory subunit** | Pfam01398 | 1e-10 |  |  |  |
|  | Smp_026630 | **proteasome regulatory subunit** | Pfam01398 | 9e-24 |  |  |  |
|  | Smp_131660 | **proteasome regulatory subunit** | Pfam01398 | 6e-19 |  |  |  |
|  | Smp_044250 | **proteasome regulatory subunit** | Pfam01398 | 6e-14 |  |  |  |
|  | Smp_158500 | LSM-10 domain | cd01733 | 1e-19 |  | 7 | 7 |

Table S4: Serine proteases

|  |  | Serine proteases |  |  |  |  |  |
| --- | --- | --- | --- | --- | --- | --- | --- |
|  |  |  |  |  |  |  |  |
| Protease family | Gene ID | Conserved domains present | Domain family | E val | Signal sequence | #TMMHM helices | # TUP helices |
| S1A | Smp_159420 | **LDLa** | 00112 | 2e-08 | 22 |  |  |
|  | Smp_002150 | trypsin-like serine protease | cd00190 | 1e-46 | 25 |  | 2 |
|  | Smp_094810 | **cyclophilin** | 01926 | 9e-74 |  |  |  |
|  | Smp_040790 | **cyclophilin** | 01926 | 8e-71 | 24 |  | 1 |
|  | Smp_159680 | **cyclophilin** | 01926 | 8e-67 |  |  |  |
|  | Smp_069160 | **cyclophilin** | 01926 | 6e-67 |  |  |  |
|  | Smp_173280 | **cyclophilin** | 01926 | 3e-74 |  |  | 7 |
|  | Smp_119130 | trypsin-like serine protease | cd00190 | 1e-14 | 25 |  | 1 |
|  | Smp_194090 | trypsin-like serine protease | cd00190 | 9e-38 |  |  |  |
|  | Smp_006510 | trypsin-like serine protease | cd00190 | 1e-20 | 25 |  | 2 |
|  | Smp_006520 | trypsin-like serine protease | cd00190 | 6e-17 | 22 | 1 |  |
|  | Smp_112090 | trypsin-like serine protease | cd00190 | 3e-20 | 25 |  | 2 |
|  | Smp_030350 | trypsin-like serine protease | cd00190 | 1e-62 | 28 |  |  |
|  | Smp_129230 | trypsin-like serine protease | cd00190 | 2e-51 |  |  |  |
|  | Smp_103680 | trypsin-like serine protease | cd00190 | 1e-63 |  |  |  |
| S1B | Smp_141070 | PDZ C-terminal processing protease | cd00988 | 4e-11 |  |  |  |
|  | Smp_162090 | trypsin | pfam00089 | 2e-13 |  |  | 6 |
|  | Smp_068530 | PDZ C-terminal processing protease | cd00988 | 3e-05 |  |  |  |
| S8A | Smp_071380 | protease associated domain | cd02123 | 9e-28 | 21 | 1 | 2 |
|  | Smp_160920 | protease associated domain | cd02123 | 7e-28 | 21 | 1 | 2 |
|  | Smp_154140 | subtilase serine protease | pfam00082 | 5e-23 |  |  | 3 |
|  | Smp_131220 | subtilase serine protease | pfam00082 | 5e-16 |  |  |  |
| S8B | Smp_160240 | subtilase serine protease | pfam00082 | 1e-51 |  |  | 1 |
|  | Smp_144080 | subtilase serine protease | pfam00082 | 1e-68 | 19 | 1 | 4 |
|  | Smp_149400 | subtilase serine protease | pfam00082 | 4e-50 |  |  |  |
| S9A | Smp_130310 | prolyl oligopeptidase | pfam00326 | 2e-31 |  |  | 6 |
|  | Smp_011590 | prolyl oligopeptidase | pfam00326 | 4e-35 |  |  | 2 |
| S9B | Smp_153060 | prolyl oligopeptidase  dipeptidyl peptidase IV | pfam00326  pfam00930 | 3e-40  6e-41 |  |  |  |
|  | Smp_057530 | prolyl oligopeptidase  dipeptidyl peptidase IV | pfam00326  pfam00930 | 5e-41  2e-41 |  | 1 | 1 |
|  | Smp_127840 | prolyl oligopeptidase  dipeptidyl peptidase IV | pfam00326  pfam00930 | 4e-21  3e-24 |  |  | 2 |
| S9C | Smp_164520 | dipeptidyl aminopeptidase | COG1506 | 4e-05 |  |  | 1 |
|  | Smp_146670 | prolyl oligopeptidase | pfam00326 | 9e-24 |  |  | 2 |
| S9X | Smp_027000 | dipeptidyl aminopeptidase | COG1506 | 3e-05 |  |  | 1 |
|  | Smp_138190 | dipeptidyl aminopeptidase | COG1506 | 2e-06 |  | 1 | 2 |
|  | Smp_000130 | **anhydrolase3** | pfam07859 | 3e-32 |  |  | 4 |
|  | Smp_038830 | dipeptidyl aminopeptidase | COG1506 | 9e-08 |  |  |  |
|  | Smp_125350 | **esterase/lipase** | cd00312 | 9e-51 | 26 |  |  |
|  | Smp_025160 | **anhydrolase2** | pfam02230 | 1e-64 |  |  |  |
| S10 | Smp_172590 | serine carboxypeptidase | pfam00450 | 2e-92 | 24 |  | 1 |
|  | Smp_163970 | serine carboxypeptidase | pfam00450 | 1e-94 | 21 |  | 2 |
| S12 | Smp_037900 | **beta-lactamase** | pfam00144 | 5e-36 |  |  | 3 |
|  | Smp_032250 | **beta-lactamase** | pfam00144 | 4e-13 |  |  |  |
| S14 | Smp_133200 | CLP protease | pfam00574 | 3e-67 |  |  | 4 |
| S16 | Smp_096360 | **replication factor C** | pfam08542 | 5e-13 |  |  |  |
|  | Smp_140530 | **replication factor C** | pfam08542 | 1e-21 |  |  |  |
|  | Smp_126490 | LonC protease | pfam05362 | 4e-57 |  |  | 1 |
| S26A | Smp_018430 | LepB signal peptidase | COG0681 | 3e-06 |  |  |  |
| S26B | Smp_031730 | LepB signal peptidase | COG0681 | 6e-05 |  | 1 | 2 |
| S28 | Smp_002600 | serine carboxypeptidase | pfam05577 | 1e-68 | 17 |  | 4 |
|  | Smp_071610 | serine carboxypeptidase | pfam05577 | 2e-74 | 24 |  | 2 |
| S33 | Smp_146180 | **ab-hydrolipase** | pfam04083 | 2e-15 |  | 1 | 2 |
|  | Smp_011000 | **ab-hydrolipase** | pfam04083 | 9e-15 |  |  |  |
|  | Smp_149920 | complex I intermediate assc. protein | pfam08547 | 9e-17 |  |  | 3 |
| S41A | Smp_155450 | PDZ C-terminal processing protease | cd00988 | 1e-10 |  |  |  |
| S54 | Smp_032420 | **none** | na | na |  | 3 | 3 |
|  | Smp_020090 | rhomboid | pfam01694 | 8e-18 |  | 7 | 8 |
|  | Smp_008620 | rhomboid | pfam01694 | 2e-20 |  | 5 | 5 |
| S58 | Smp_158330 | **chromosome segregation ATPase** | COG1196 | 2e-05 |  |  |  |
| S59 | Smp_027890 | nucleoporin autopeptidase | pfam04096 | 8e-50 |  |  |  |
| S63 | Smp_058380 | **latrophilin/CL1-like** | pfam01825 | 7e-10 |  | 7 | 7 |
|  |  |  |  |  |  |  |  |
|  |  |  |  |  |  |  |  |
|  |  |  |  |  |  |  |  |
|  |  |  |  |  |  |  |  |
|  |  |  |  |  |  |  |  |
|  |  |  |  |  |  |  |  |
|  |  |  |  |  |  |  |  |
|  |  |  |  |  |  |  |  |
|  |  |  |  |  |  |  |  |

Table S5 Threonine proteases

|  |  | Threonine proteases |  |  |  |  |  |
| --- | --- | --- | --- | --- | --- | --- | --- |
| Protease family | Gene ID | Conserved domains present | Domain family | E val | Signal sequence | #TMMHM helices | # TUP helices |
| T1A | Smp_076230 | proteasome alpha type 7 | cd03755 | 1e-97 |  |  |  |
|  | Smp_073410 | proteasome beta type 7 | cd03763 | 2e-79 |  |  |  |
|  | Smp_092280 | proteasome alpha type 3 | cd03751 | 2e-99 |  |  |  |
|  | Smp_164840 | proteasome beta type 5 | cd03761 | 2e-91 |  |  | 3 |
|  | Smp_067890 | proteasome alpha type 2 | cd03750 | 9e-105 |  |  |  |
|  | Smp_025800 | proteasome beta type 1 | cd03757 | 1e-80 |  |  |  |
|  | Smp_070930 | proteasome alpha type 4 | cd03752 | 2e-97 |  |  |  |
|  | Smp_056500 | proteasome beta type 4 | cd03760 | 4e-80 |  |  |  |
|  | Smp_170730 | proteasome alpha type 1 | cd03749 | 8e-93 |  |  | 1 |
|  | Smp_034490 | proteasome beta type 6 | cd03762 | 9e-79 |  |  |  |
|  | Smp_074500 | ntn-hydrolase | cl00467 | 5e-51 |  |  | 1 |
|  | Smp_121430 | proteasome beta type 3 | cd03759 | 7e-87 |  |  | 1 |
|  | Smp_130110 | proteasome alpha type 6 | cd03754 | 2e-97 |  |  |  |
| T2 | Smp_011150 | taspase | cd04514 | 9e-80 |  |  |  |
|  | Smp_173480 | glycosyl asparginase | cd04513 | 2e-86 | 23 |  | 1 |
| T3 | Smp_089100 | gamma glutamyltranspeptidase | cl08040 | 2e-39 |  | 1 | 2 |
|  |  |  |  |  |  |  |  |
|  |  |  |  |  |  |  |  |
