# Supplementary material for: Analysis of regulatory protease sequences identified through bioinformatic data mining of the Schistosoma mansoni genome
Source: BMC Genomics. 2009 Oct 21;10:488. doi: 10.1186/1471-2164-10-488 (PMC2772863; doi:10.1186/1471-2164-10-488)
Supplement: Additional file 2 — KAAS analysis: KEGG pathway assignment and KEGG orthology number (KO number) of each S. mansoni protease. Bioinformatic analysis using the Kyoto Encyclopedia of Genes and Genomes used to predict probable functions and the cellular processes for S. mansoni proteases, based on orthologous relationships of proteases for which function is clearly established in other species. [file 1471-2164-10-488-S2.DOC]

Additional file 2: KAAS analysis: KEGG pathway assignment and KEGG orthology number (KO number) of each *S. mansoni* protease.

01100 Metabolism

**01101 Carbohydrate Metabolism**

[00530](http://www.genome.jp/kegg-bin/mark_pathway_www?@ko00530/reference%3Dwhite/default%3D%23bfffbf/K00820/K01443) Aminosugars metabolism (2)

**01102 Energy Metabolism**

[00190](http://www.genome.jp/kegg-bin/mark_pathway_www?@ko00190/reference%3Dwhite/default%3D%23bfffbf/K00415) Oxidative phosphorylation (1)

**01103 Lipid Metabolism**

[00561](http://www.genome.jp/kegg-bin/mark_pathway_www?@ko00561/reference%3Dwhite/default%3D%23bfffbf/K01046) Glycerolipid metabolism (1)

[00564](http://www.genome.jp/kegg-bin/mark_pathway_www?@ko00564/reference%3Dwhite/default%3D%23bfffbf/K06130) Glycerophospholipid metabolism (1)

[00590](http://www.genome.jp/kegg-bin/mark_pathway_www?@ko00590/reference%3Dwhite/default%3D%23bfffbf/K00681/K01254) Arachidonic acid metabolism (2)

**01104 Nucleotide Metabolism**

[00230](http://www.genome.jp/kegg-bin/mark_pathway_www?@ko00230/reference%3Dwhite/default%3D%23bfffbf/K01951) Purine metabolism (1)

[00240](http://www.genome.jp/kegg-bin/mark_pathway_www?@ko00240/reference%3Dwhite/default%3D%23bfffbf/K11540/K01937/K01464) Pyrimidine metabolism (3)

**01105 Amino Acid Metabolism**

[00251](http://www.genome.jp/kegg-bin/mark_pathway_www?@ko00251/reference%3Dwhite/default%3D%23bfffbf/K11540/K00820/K01951) Glutamate metabolism (3)

[00252](http://www.genome.jp/kegg-bin/mark_pathway_www?@ko00252/reference%3Dwhite/default%3D%23bfffbf/K11540) Alanine and aspartate metabolism (1)

[00220](http://www.genome.jp/kegg-bin/mark_pathway_www?@ko00220/reference%3Dwhite/default%3D%23bfffbf/K01436) Urea cycle and metabolism of amino groups (1)

**01106 Metabolism of Other Amino Acids**

[00410](http://www.genome.jp/kegg-bin/mark_pathway_www?@ko00410/reference%3Dwhite/default%3D%23bfffbf/K01464) beta-Alanine metabolism (1)

[00430](http://www.genome.jp/kegg-bin/mark_pathway_www?@ko00430/reference%3Dwhite/default%3D%23bfffbf/K00681) Taurine and hypotaurine metabolism (1)

[00450](http://www.genome.jp/kegg-bin/mark_pathway_www?@ko00450/reference%3Dwhite/default%3D%23bfffbf/K00681) Selenoamino acid metabolism (1)

[00460](http://www.genome.jp/kegg-bin/mark_pathway_www?@ko00460/reference%3Dwhite/default%3D%23bfffbf/K00681) Cyanoamino acid metabolism (1)

[00480](http://www.genome.jp/kegg-bin/mark_pathway_www?@ko00480/reference%3Dwhite/default%3D%23bfffbf/K00681/K01255/K11140/K01256) Glutathione metabolism (4)

**01107 Glycan Biosynthesis and Metabolism**

[00512](http://www.genome.jp/kegg-bin/mark_pathway_www?@ko00512/reference%3Dwhite/default%3D%23bfffbf/K00710) O-Glycan biosynthesis (1)

[00563](http://www.genome.jp/kegg-bin/mark_pathway_www?@ko00563/reference%3Dwhite/default%3D%23bfffbf/K05290) Glycosylphosphatidylinositol(GPI)-anchor biosynthesis (1)

[00531](http://www.genome.jp/kegg-bin/mark_pathway_www?@ko00531/reference%3Dwhite/default%3D%23bfffbf/K01197) Glycosaminoglycan degradation (1)

[00511](http://www.genome.jp/kegg-bin/mark_pathway_www?@ko00511/reference%3Dwhite/default%3D%23bfffbf/K01444) Other glycan degradation (1)

[01030](http://www.genome.jp/kegg-bin/mark_pathway_www?@ko01030/reference%3Dwhite/default%3D%23bfffbf/K00710) Glycan structures - Biosynthesis 1 (1)

[01031](http://www.genome.jp/kegg-bin/mark_pathway_www?@ko01031/reference%3Dwhite/default%3D%23bfffbf/K05290) Glycan structures - Biosynthesis 2 (1)

[01032](http://www.genome.jp/kegg-bin/mark_pathway_www?@ko01032/reference%3Dwhite/default%3D%23bfffbf/K01444/K01197) Glycan structures - Degradation (2)

**01109 Metabolism of Cofactors and Vitamins**

[00770](http://www.genome.jp/kegg-bin/mark_pathway_www?@ko00770/reference%3Dwhite/default%3D%23bfffbf/K01464) Pantothenate and CoA biosynthesis (1)

**01110 Biosynthesis of Secondary Metabolites**

[00960](http://www.genome.jp/kegg-bin/mark_pathway_www?@ko00960/reference%3Dwhite/default%3D%23bfffbf/K01044/K01066) Alkaloid biosynthesis II (2)

**01111 Xenobiotics Biodegradation and Metabolism**

[00623](http://www.genome.jp/kegg-bin/mark_pathway_www?@ko00623/reference%3Dwhite/default%3D%23bfffbf/K01066) 2,4-Dichlorobenzoate degradation (1)

[00983](http://www.genome.jp/kegg-bin/mark_pathway_www?@ko00983/reference%3Dwhite/default%3D%23bfffbf/K01951/K01044/K03927/K01464) Drug metabolism - other enzymes (4)

01120 Genetic Information Processing

**01121 Transcription**

[03022](http://www.genome.jp/kegg-bin/mark_pathway_www?@ko03022/reference%3Dwhite/default%3D%23bfffbf/K03128) Basal transcription factors (1)

**01123 Folding, Sorting and Degradation**

[03060](http://www.genome.jp/kegg-bin/mark_pathway_www?@ko03060/reference%3Dwhite/default%3D%23bfffbf/K03100) Protein export (1)

[04140](http://www.genome.jp/kegg-bin/mark_pathway_www?@ko04140/reference%3Dwhite/default%3D%23bfffbf/K08342) Regulation of autophagy (1)

[03050](http://www.genome.jp/kegg-bin/mark_pathway_www?@ko03050/reference%3Dwhite/default%3D%23bfffbf/K03038/K03061/K03062/K03066/K02730/K02726/K02728/K02731/K02725/K02727/K02738/K02739/K02735/K02734/K02737/K02732/K02736) Proteasome (17)

**01124 Replication and Repair**

[03030](http://www.genome.jp/kegg-bin/mark_pathway_www?@ko03030/reference%3Dwhite/default%3D%23bfffbf/K10755) DNA replication (1)

[03420](http://www.genome.jp/kegg-bin/mark_pathway_www?@ko03420/reference%3Dwhite/default%3D%23bfffbf/K10755) Nucleotide excision repair (1)

[03430](http://www.genome.jp/kegg-bin/mark_pathway_www?@ko03430/reference%3Dwhite/default%3D%23bfffbf/K10755) Mismatch repair (1)

[03450](http://www.genome.jp/kegg-bin/mark_pathway_www?@ko03450/reference%3Dwhite/default%3D%23bfffbf/K10884) Non-homologous end-joining (1)

01130 Environmental Information Processing

**01132 Signal Transduction**

[04010](http://www.genome.jp/kegg-bin/mark_pathway_www?@ko04010/reference%3Dwhite/default%3D%23bfffbf/K02187) MAPK signaling pathway (1)

[04310](http://www.genome.jp/kegg-bin/mark_pathway_www?@ko04310/reference%3Dwhite/default%3D%23bfffbf/K04496/K04505) Wnt signaling pathway (2)

[04330](http://www.genome.jp/kegg-bin/mark_pathway_www?@ko04330/reference%3Dwhite/default%3D%23bfffbf/K06059/K04505/K04496) Notch signaling pathway (3)

[04340](http://www.genome.jp/kegg-bin/mark_pathway_www?@ko04340/reference%3Dwhite/default%3D%23bfffbf/K02218) Hedgehog signaling pathway (1)

[04350](http://www.genome.jp/kegg-bin/mark_pathway_www?@ko04350/reference%3Dwhite/default%3D%23bfffbf/K04659) TGF-beta signaling pathway (1)

**01133 Signaling Molecules and Interaction**

[04080](http://www.genome.jp/kegg-bin/mark_pathway_www?@ko04080/reference%3Dwhite/default%3D%23bfffbf/K01315/K01312) Neuroactive ligand-receptor interaction (2)

[04512](http://www.genome.jp/kegg-bin/mark_pathway_www?@ko04512/reference%3Dwhite/default%3D%23bfffbf/K04659) ECM-receptor interaction (1)

01140 Cellular Processes

**01142 Cell Growth and Death**

[04110](http://www.genome.jp/kegg-bin/mark_pathway_www?@ko04110/reference%3Dwhite/default%3D%23bfffbf/K02365) Cell cycle (1)

[04111](http://www.genome.jp/kegg-bin/mark_pathway_www?@ko04111/reference%3Dwhite/default%3D%23bfffbf/K02365) Cell cycle - yeast (1)

[04210](http://www.genome.jp/kegg-bin/mark_pathway_www?@ko04210/reference%3Dwhite/default%3D%23bfffbf/K04398/K02187/K04397) Apoptosis (3)

[04115](http://www.genome.jp/kegg-bin/mark_pathway_www?@ko04115/reference%3Dwhite/default%3D%23bfffbf/K04398/K02187) p53 signaling pathway (2)

**01143 Cell Communication**

[04510](http://www.genome.jp/kegg-bin/mark_pathway_www?@ko04510/reference%3Dwhite/default%3D%23bfffbf/K04659) Focal adhesion (1)

**01144 Endocrine System**

[04910](http://www.genome.jp/kegg-bin/mark_pathway_www?@ko04910/reference%3Dwhite/default%3D%23bfffbf/K07188) Insulin signaling pathway (1)

[04614](http://www.genome.jp/kegg-bin/mark_pathway_www?@ko04614/reference%3Dwhite/default%3D%23bfffbf/K11140/K01287/K01389/K01392) Renin - angiotensin system (4)

**01145 Immune System**

[04640](http://www.genome.jp/kegg-bin/mark_pathway_www?@ko04640/reference%3Dwhite/default%3D%23bfffbf/K01389/K11140) Hematopoietic cell lineage (2)

[04610](http://www.genome.jp/kegg-bin/mark_pathway_www?@ko04610/reference%3Dwhite/default%3D%23bfffbf/K01315) Complement and coagulation cascades (1)

[04620](http://www.genome.jp/kegg-bin/mark_pathway_www?@ko04620/reference%3Dwhite/default%3D%23bfffbf/K04398) Toll-like receptor signaling pathway (1)

[04650](http://www.genome.jp/kegg-bin/mark_pathway_www?@ko04650/reference%3Dwhite/default%3D%23bfffbf/K02187) Natural killer cell mediated cytotoxicity (1)

[04612](http://www.genome.jp/kegg-bin/mark_pathway_www?@ko04612/reference%3Dwhite/default%3D%23bfffbf/K01369/K01363/K01365) Antigen processing and presentation (3)

**01148 Development**

[04360](http://www.genome.jp/kegg-bin/mark_pathway_www?@ko04360/reference%3Dwhite/default%3D%23bfffbf/K07528) Axon guidance (1)

01160 Human Diseases

**01161 Cancers**

[05210](http://www.genome.jp/kegg-bin/mark_pathway_www?@ko05210/reference%3Dwhite/default%3D%23bfffbf/K02187) Colorectal cancer (1)

[05220](http://www.genome.jp/kegg-bin/mark_pathway_www?@ko05220/reference%3Dwhite/default%3D%23bfffbf/K04496) Chronic myeloid leukemia (1)

**01163 Neurodegenerative Diseases**

[05010](http://www.genome.jp/kegg-bin/mark_pathway_www?@ko05010/reference%3Dwhite/default%3D%23bfffbf/K06704/K06059/K07747/K04505/K01408/K01389/K00415/K04398/K02187/K04397) Alzheimer's disease (10)

[05012](http://www.genome.jp/kegg-bin/mark_pathway_www?@ko05012/reference%3Dwhite/default%3D%23bfffbf/K00415/K02187) Parkinson's disease (2)

[05014](http://www.genome.jp/kegg-bin/mark_pathway_www?@ko05014/reference%3Dwhite/default%3D%23bfffbf/K02187) Amyotrophic lateral sclerosis (ALS) (1)

[05016](http://www.genome.jp/kegg-bin/mark_pathway_www?@ko05016/reference%3Dwhite/default%3D%23bfffbf/K04398/K02187/K00415) Huntington's disease (3)

**01164 Metabolic Disorders**

[04940](http://www.genome.jp/kegg-bin/mark_pathway_www?@ko04940/reference%3Dwhite/default%3D%23bfffbf/K01294) Type I diabetes mellitus (1)

**01165 Infectious Diseases**

[05120](http://www.genome.jp/kegg-bin/mark_pathway_www?@ko05120/reference%3Dwhite/default%3D%23bfffbf/K06059/K06704/K02187) Epithelial cell signaling in Helicobacter pylori infection (3)

| Protease and orthology |
| --- |
| Smp_132480 K01379 |
| Smp_136830.2 K01379 |
| Smp_013040.2 K01379 |
| Smp_136730 K01379 |
| Smp_175560 K07747 |
| Smp_190380 |
| Smp_154310 |
| Smp_153960 K04505 |
| Smp_154770 K09596 |
| Smp_155880 K07575 |
| Smp_121030 |
| Smp_157090 K01365 |
| Smp_179950 K01363 |
| Smp_158420 K01363 |
| Smp_103610 K01363 |
| Smp_067060 K01363 |
| Smp_034410.1 K01373 |
| Smp_019030 K01275 |
| Smp_141610 K01363 |
| Smp_139160 K01365 |
| Smp_193000 K01365 |
| Smp_149730 K01365 |
| Smp_085010 K01363 |
| Smp_105370 K01363 |
| Smp_181030 K01363 |
| Smp_139240 K01365 |
| Smp_089460.2 K08585 |
| Smp_003980 K08585 |
| Smp_137410 K08578 |
| Smp_159550 K08574 |
| Smp_130640 |
| Smp_167480.1 K08576 |
| Smp_083530 K08576 |
| Smp_157500 K08585 |
| Smp_147920 K08588 |
| Smp_083200.1 K05610 |
| Smp_168800.2 K05609 |
| Smp_011230 K05290 |
| Smp_075800.1 K01369 |
| Smp_032000 K04489 |
| Smp_028500 K02187 |
| Smp_141270 K04398 |
| Smp_172010 K04397 |
| Smp_140810 K01304 |
| Smp_021300 K11848 |
| Smp_047360.1 |
| Smp_125860 K11858 |
| Smp_046430 K11855 |
| Smp_074200 K11844 |
| Smp_175520 K11833 |
| Smp_000710 K11842 |
| Smp_122440 K11842 |
| Smp_089180 K11838 |
| Smp_074400 K11366 |
| Smp_123630 K11834 |
| Smp_058560 K11840 |
| Smp_153690 K11840 |
| Smp_017890 K01072 |
| Smp_128770 K11835 |
| Smp_069960 K11836 |
| Smp_152000 K11839 |
| Smp_162200.2 K11844 |
| Smp_131570 K11840 |
| Smp_168120 K02218 |
| Smp_038140 |
| Smp_005280 K11841 |
| Smp_122960.1 |
| Smp_063830 K01951 |
| Smp_134650 K01937 |
| Smp_136260 K00820 |
| Smp_033260.1 K08592 |
| Smp_159120 K08596 |
| Smp_121810 |
| Smp_121890 K08597 |
| Smp_167810 K02365 |
| Smp_039820.2 K08342 |
| Smp_124830.1 K08342 |
| Smp_082030 K03152 |
| Smp_052350 K11862 |
| Smp_173030 K11140 |
| Smp_128960 K03128 |
| Smp_007550 K01254 |
| Smp_091470 K08776 |
| Smp_174530 K01256 |
| Smp_075220.1 K01410 |
| Smp_029500 K01392 |
| Smp_029470 K01392 |
| Smp_127030 K01404 |
| Smp_171330 |
| Smp_090100 |
| Smp_153930 K01404 |
| Smp_135530 K01404 |
| Smp_173070 K01404 |
| Smp_167090 K01404 |
| Smp_171340 |
| Smp_090110 |
| Smp_167120 K01404 |
| Smp_167100 K01404 |
| Smp_162300 |
| Smp_145930 K07995 |
| Smp_134430 K09608 |
| Smp_047460 K08076 |
| Smp_160620.1 K08608 |
| Smp_175340.1 K06059 |
| Smp_171690 K04659 |
| Smp_145900 K06704 |
| Smp_124500 K01417 |
| Smp_146730 K08619 |
| Smp_163920 |
| Smp_194190 K01389 |
| Smp_122850 |
| Smp_159370 K01415 |
| Smp_170500 K01415 |
| Smp_170470 |
| Smp_181710 |
| Smp_157440 K01389 |
| Smp_122860 |
| Smp_171100 K01389 |
| Smp_157400 K08635 |
| Smp_173160 K01415 |
| Smp_122450 K08635 |
| Smp_000760 K08635 |
| Smp_088270 K01290 |
| Smp_159890 K07752 |
| Smp_167640 |
| Smp_181340 |
| Smp_125080 |
| Smp_127610 |
| Smp_142190 K01294 |
| Smp_082250 |
| Smp_128100 K01408 |
| Smp_061510 K01411 |
| Smp_156960.2 K01408 |
| Smp_155230.1 K01411 |
| Smp_094050.2 K01412 |
| Smp_079450 K00415 |
| Smp_009650.1 K01412 |
| Smp_146140 K06972 |
| Smp_155220 K01411 |
| Smp_030000 K01255 |
| Smp_083870.2 K01255 |
| Smp_138380 K01255 |
| Smp_159960.1 K01267 |
| Smp_019630 K01436 |
| Smp_111420 K01258 |
| Smp_125030 K01409 |
| Smp_142010.2 K01265 |
| Smp_159490 K00710 |
| Smp_011120 K01265 |
| Smp_050070 K01262 |
| Smp_148960.2 K01262 |
| Smp_090800 K01271 |
| Smp_127540 K01197 |
| Smp_088280 |
| Smp_150690.1 |
| Smp_135510 K01301 |
| Smp_175220 |
| Smp_170400 |
| Smp_085110.2 K07528 |
| Smp_106150 K11540 |
| Smp_033660 K01443 |
| Smp_078780 K01464 |
| Smp_126390 K01464 |
| Smp_018620 K03798 |
| Smp_119310 K03066 |
| Smp_012470 K03061 |
| Smp_018240.1 |
| Smp_173840 K03062 |
| Smp_126110 K07767 |
| Smp_165550 K03798 |
| Smp_055760.1 K03798 |
| Smp_082620 K06013 |
| Smp_019010 K01277 |
| Smp_054310 K07765 |
| Smp_178770 |
| Smp_126690 |
| Smp_026630 K03038 |
| Smp_044250 K11866 |
| Smp_131660 K09613 |
| Smp_158500 K03247 |
| Smp_159420 |
| Smp_002150 K01312 |
| Smp_094810 K09564 |
| Smp_040790 K03768 |
| Smp_159680.2 K09566 |
| Smp_069160 K09566 |
| Smp_173280 K09567 |
| Smp_119130 |
| Smp_194090 K01316 |
| Smp_006510 K01345 |
| Smp_006520 |
| Smp_112090 K01345 |
| Smp_030350 K01315 |
| Smp_129230 K09634 |
| Smp_103680 K09626 |
| Smp_141070 |
| Smp_162090 K04496 |
| Smp_068530 |
| Smp_071380.2 |
| Smp_160920.1 |
| Smp_154140 K08653 |
| Smp_131220 K01280 |
| Smp_160240 K01349 |
| Smp_144080 K08654 |
| Smp_149400 K01359 |
| Smp_130310 |
| Smp_011590.1 K01322 |
| Smp_153060 K01278 |
| Smp_057530 |
| Smp_127840 K01281 |
| Smp_164520 |
| Smp_146670 K10884 |
| Smp_027000 |
| Smp_138190 K01044 |
| Smp_000130.1 K07188 |
| Smp_038830 |
| Smp_125350 K03927 |
| Smp_025160.1 K06130 |
| Smp_172590 K01287 |
| Smp_163970 K01287 |
| Smp_037900 |
| Smp_032250 |
| Smp_133200 K01358 |
| Smp_096360 K10755 |
| Smp_140530 K10755 |
| Smp_126490 K08675 |
| Smp_018430 K09647 |
| Smp_031730 K03100 |
| Smp_002600.1 K01285 |
| Smp_071610 K01276 |
| Smp_146180 K01046 |
| Smp_011000 K01046 |
| Smp_149920 K01066 |
| Smp_155450 |
| Smp_032420 K09650 |
| Smp_020090 |
| Smp_008620 |
| Smp_158330 |
| Smp_027890 |
| Smp_058380 K04592 |
| Smp_076230 K02731 |
| Smp_073410 K02739 |
| Smp_092280 K02727 |
| Smp_164840 K02737 |
| Smp_067890 K02726 |
| Smp_025800 K02732 |
| Smp_070930 K02728 |
| Smp_056500 K02736 |
| Smp_170730 K02725 |
| Smp_034490 K02738 |
| Smp_074500.1 K02734 |
| Smp_121430.1 K02735 |
| Smp_130110 K02730 |
| Smp_011150 K08657 |
| Smp_173480 K01444 |
| Smp_089100 K00681 |
