# Supplementary material for: Analysis of regulatory protease sequences identified through bioinformatic data mining of the Schistosoma mansoni genome
Source: BMC Genomics. 2009 Oct 21;10:488. doi: 10.1186/1471-2164-10-488 (PMC2772863; doi:10.1186/1471-2164-10-488)
Supplement: Additional file 3 — Figures of catalytic motifs and active sites of selected protease families. A series of six figures, each of which shows a partial sequence alignment of a family of proteases for several species. Highlighted are catalytic motifs which are directly involved in catalysis in that family of proteases, and the active site itself, which is essential to the proteolytic function. [file 1471-2164-10-488-S3.DOC]

Additional file 3. Figures of catalytic motifs and active sites of selected protease families

Figure S1. A22 protease family catalytic motif (active site shown in black)

| Schistosoma_155880  C_elegans_IMP3 [Q18408]  Schistosoma_154770  C_elegans_IMP2 [P49049]  Human_HM13 [Q8TCT9]  Mouse_HM13 [Q9D8V0]  Human_SPPL3 [Q8TCT6]  Mouse_SPPL3 [Q9CUS9]  C_elegans_IMP1 [Q93346]  Human_SPPL2A [Q8TCT8]  Mouse_SPPL2A [Q9JJF9]  Human_SPPL2B [Q8TCT7]  Mouse_SPPL2B [Q8BXJ5]  Human_SPPL2C [Q8IUH8]  Mouse_SPPL2B [A2A6C4]  C_elegans_SPE4 [Q01608]  C_elegans_HOP1 [O02100]  Schistosoma_153960  C_elegans_SEL12 [P52166]  Human_PSEN2 [P49810]  Mouse_PSEN2 [Q61144]  Human_PSEN1 [P49768]  Mouse_PSEN1 [P49769] | ....|....|....|....|....|....|....|....|....|.... ....|....|....|....|....|....  460 470 480 490 680 690 700  LNNFLNNQNVRH----TFFINQ-----VDDLRFNRGALLNVGALESLEA TLVG----GMSNSFWGWGREDDEFQIRLK  SGNWISNDILAFASIY-VVCR------IQAVSYQTAIIFVIGMSLFDLF QSLAALDIMVPGVFLNVVLKYSSMYDTNL  IG-WPLQSVIGM------FIAVIISSALIIPSVKVGTLLFTVFMIYDIF SLLGFGDAVIPGIFIQFLAFYDAC-WRTP  ---WITNNIIGV-----SFSLGI--ERLHLASFKAGSLLLVGLFFYDIF SMLGLGDIVIPGIFIALLRRFDYRVVQTT  ---WIANNLFGL-----AFSNGV--ELLHLNNVSTGCILLGGLFIYDVF AMLGLGDVVIPGIFIALLLRFDISLKKNT  ---WIANNLFGL-----AFSNGV--ELLHLNNVSTGCILLGGLFIYDIF AMLGLGDIVIPGIFIALLLRFDISLKKNT  ---WLLMDALAM-----GLCAMI--AFVRLPSLKVSCLLLSGLLIYDVF SMLGIGDIVMPGLLLCFVLRYDNYKKQAS  ---WLLMDALAM-----GLCAMI--AFDRLPSLKVSCLLLSGLLIYDVF SMLGIGDIVMPGLLLCFVLRYDNYKKQAS  A--FILLDVINM-----ALCHVL--KCLRLPSLKWISILMLCMFVYDAF TILGLGDIVMPGYLVAHCFTMNGFSERVR  WA-WILQDILGI-----AFCNLI--KTLKLPNFKSCVILLGLLLLYDVF SILGFGDIIVPGLLIAYCRRFDVQTGSSY  WA-WILQDILGI-----AFCNLI--KTMKLPNFMSCVILLGLLLIYDVF SVLGFGDIIVPGLLIAYCRRFDVQTGSSI  WA-WVLQDALGI-----AFCYML--KTIRLPTFKACTLLLLVLFLYDIF SLLGFGDILVPGLLVAYCHRFDIQVQSSR  WA-WVLQDTLGI-----AFCYML--KTIRLPTFKACTLLLLVLFIYDIF SLLGFGDILVPGLLVAYCHRFDIQVQSSR  WA-WLLQDTLGI-----SYCFVL--HRVRLPTLKNCSSFLLALLAFDVF SILGFGDIVVPGFLVAYCCRFDVQVCSRQ  WA-WLLQDTLGV-----AYCFVL--RRVRLPTFKNCTLFLLALLAFDVF SILGFGDIVVPGFLVAYCHRFDMQVQSRQ  KTTWFVLWIVLFWDLFAVLAMGPLKKVQEKASDYSKCVLNLIMFSANEK LRLGFGDFVFYSLLIGQAAASGCPFAV--  WTVWMALTAISFWDIVAVLTCGPLKMLVETANRRGDDKFPAILYNSSSY IRLGMGDFVFYSLMLGNTVQT--CPLPTV  WTCWLVLAALSVWDIVAVLCNGPLRLLVEMAHERQQPLFPALLYSTTTV VKLGLGDFVFYSLLIGRATLD--GDAVTV  WTVWFVLFVISVWDLVAVLTKGPLRYLVETAQERNEPIFPALIYSSGVI VKLGLGDFIFYSVLLGKASSY--FDWNTT  WSAWVILGAISVYDLVAVLCKGPLRMLVETAQERNEPIFPALIYSSAMV VKLGLGDFIFYSVLVGKAAATGSGDWNTT  WSAWVILGAISVYDLVAVLCKGPLRMLVETAQERNEPIFPALIYSSAMV VKLGLGDFIFYSVLVGKAAATGNGDWNTT  WTAWLILAVISVYDLVAVLCKGPLRMLVETAQERNETLFPALIYSSTMV VKLGLGDFIFYSVLVGKASATASGDWNTT  WTAWLILAVISVYDLVAVLCKGPLRMLVETAQERNETLFPALIYSSTMV VKLGLGDFIFYSVLVGKASATASGDWNTT |
| --- | --- |

Figure S2. C14 protease family (caspase) catalytic motif (active site shown in black)

| Fruit fly_DRONC [Q9XYf4]  Human_CASP1 [P29466]  Mouse_CASP1 [P2952]  Mouse_CASP12 [O08736]  Mouse_CASP4 [P70343]  Mouse_CASP11 [NP_031635]  Human_CASP4 [P49662]  Human_CASP5 [P51878]  Human_CASP2 [P42575]  Mouse_CASP2 [P29594]  Schistosoma_141270  Schistosoma_032000  Human_CASP14 [P31994]  Fruit fly_DREDD [ABI74776]  A_aegyptii_DREDD [Q8IRY7]  Fruit fly_DAMM [Q9NBW3]  Fruti fly_STRICTA [Q7KHK9]  Human_CASP10 [Q92851]  Human_CASP9 [P55211]  Mouse_CASP9 [NP_056458]  Human_CASP8 [Q14790]  Mouse_CASP8 [O89110]  Schistosoma_172010  Fruit fly_CASP1 [NP_524551]  Fruit fly_DC[1 [NP_426974]  Human_CASP6 [P55212]  Mouse_CASP6 [O08738]  Schistosoma_028500  Human_CASP7 [P55210]  Mouse_CASP7 [P77864]  Human_CASP3 [P42574]  Mouse_CASP3 [70677]  C_elegans_CED3 [P42573]  C_elegans_CSP1 [O18203]  C_elegans_CSP2 [Q9ZP5]  C_elegans_CSP3 [Q9XWE0] | |....|....|....|....|....|....|....|....|....|....|....|....|....|....|  135 145 155 165 175 185 195  TECFVMVLMTHGNSVE---GKEKVEFCDGSVV-DMQKIKDHFQTAKCPYLVNKPKVLMFPFCRGDEYDL-G  SDSTFLVFMSHGIREG---ICGKKHSEQVPDILQLNAIFNMLNTKNCPSLKDKPKVIIIQACRGDSPGV--  SDSTFLVFMSHGIQEG---ICGTTYSNEVSDILKVDTIFQMMNTLKCPSLKDKPKVIIIQACRGEKQGV-V  SDSTFLVFMSHGILEG---ICGVKHRNKKPDVLHDDTIFKIFNNSNCRSLRNKPKILIMQACRGRYNGT--  SDSTFLVLMSHGTLHG---ICGTMHSEKTPDVLQYDTIYQIFNNCHCPGLRDKPKVIIVQACRGGNSGE--  SDSTFLVLMSHGTLHG---ICGTMHSEKTPDVLQYDTIYQIFNNCHCPGLRDKPKVIIVQACRGGNSGE--  SDSTFLVLMSHGILEG---ICGTVHDEKKPDVLLYDTIFQIFNNRNCLSLKDKPKVIIVQACRGANRGE--  SDSTFLVLMSHGILEG---ICGTAHKKKKPDVLLYDTIFQIFNNRNCLSLKDKPKVIIVQACRGEKHGE--  TDSCIVALLSHGVE-------GAIYGVDGKLL-QLQEVFQLFDNANCPSLQNKPKMFFIQACRGDETDR-G  TDSCVVALLSHGVE-------GGIYGVDGKLL-QLQEVFRLFDNANCPSLQNKPKMFFIQACRGDETDR-G  YDCLVICLMSHGTI-------GRIYGVDGNSL-SIHELTSIFTADNCPSLAGKPKLFFIQACRGEDYQK-G  VHAAVLIILAHGLE-------HHIIASDGIHV-SIDELVGCFTNKRCPLLAGKPKLILIQACRGEERNHNG  VSCAFVVLMAHGRE-------GFLKGEDGEMV-KLENLFEALNNKNCQALRAKPKVYIIQACRGEQRDP-G  -DSLVVFILSHGFE-------EAVYASNSIAM-KITDIEDLLCSYD--TLYYKPKLLIIQACQEKLVHK--  HCSLVICLLSHGQE-------GKVYGSNSIPV-SVKAIERKMAARK---LTGKPKLLFVQACQGSGLQT-A  -AGFVLFILSHGDRK------EKILACDHREY-HLDD-DVLFPLFRNPTLSGKPKILIVQACKGPLRAD--  KSALVLVILSHGTRH------DQIAAKDDDYSLDDDVVFPILRNR---TLKDKPKLIFVQACKGDCQLG-G  GDCFVFCILTHGRF-------GAVYSSDEALI-PIREIMSHFTALQCPRLAEKPKLFFIQACQGEEIQP-S  LDCCVVVILSHGCQASHLQFPGAVYGTDGCPV-SVEKIVNIFNGTSCPSLGGKPKLFFIQACGGEQKDH-G  LDCFVVVILSHGCQASHLQFPGAVYGTDGCSV-SIEKIVNIFNGSGCPSLGGKPKLFFIQACGGEQKDH-G  MDCFICCILSHGDK-------GIIYGTDGQEA-PIYELTSQFTGLKCPSLAGKPKVFFIQACQGDNYQK-G  KDCFICCILSHGDK-------GVVYGTDGKEA-SIYDLTSYFTGSKCPSLSGKPKIFFIQACQGSNFQK-G  HDCFACVILSHGDEG------GLIYATDGSI--PVDRIIAPFRGDQCLDLRGKPKLFFIQACRGMALDD-G  SDCILVAILSHGEM-------GYIYAKDTQY--KLDNIWSFFTANHCPSLAGKPKLFFIQACQGDRLDG-G  NDCLAVAILSHGEH-------GYLYAKDTQY--KLDNIWHYFTATFCPSLAGKPKLFFIQACQGDRLDG-G  ADCFVCVFLSHGEG-------NHIYAYDAKI--EIQTLTGLFKGDKCHSLVGKPKIFIIQACRGNQHDVPV  ADCFICVFLSHGEG-------NHVYAYDAKI--EIQTLTGLFKGDKCQSLVGKPKIFIIQACRGSQHDVPV  YDSFIFVMLSHGDN-------NIIYANDGEVLTSY--IMAFFRGDRCPSLIAKPKLFFFQACRGAAFDK-G  AACFACILLSHGEE-------NVIYGKDGVT--PIKDLTAHFRGDRCKTLLEKPKLFFIQACRGTELDD-G  SACFACVLLSHGEE-------DLIYGKDGVT--PIKDLTAHFRGDRCKTLLEKPKLFFIQACRGTELDD-G  RSSFVCVLLSHGEE-------GIIFGTNGPV--DLKKITNFFRGDRCRSLTGKPKLFIIQACRGTELDC-G  RSSFVCVILSHGDE-------GVIYGTNGPV--ELKKLTSFFRGDYCRSLTGKPKLFIIQACRGTELDC-G  -DSAILVILSHGEE-------NVIIGVDDIPI-STHEIYDLLNAANAPRLANKPKIVFVQACRGERRDN-G  -DSIILFLLSHGDGA------GSVFGIDDMPV-NVMEVSTYL--AYHQNLLLKPKWVAVSACRGGKLNM-G  -DSLIITIMSHGDQ-------GLLYGVDGVPV-QMLDIIDLMCTA---SLAKKPKWLMCVCCRGDRIDR-A  -------------------------------------IDNFFDRI--------PKFFQFMKSK-------- |
| --- | --- |

Figure S3. M50 protease family catalytic motifs (active site shown in black)

| Fruit fly_MBTPS2 [Q7JZ56]  C_elegans_MBTPS2 [Q9U227]  Schistosoma_054310  Mouse_MBTPS2 [Q8CHX6]  Human_MBTPS2 [O43462]  Frog_MBTPS2 [A4IGW4]  Chicken_MBTPS2 [XP_425566] | |....|....|....|....|....|....|....|....|....|....|....|....|  265 275 285 295 305 315 325  GVNLPLEEIGYYITTLVLCLVVHEMGHALAAVMEDVPVTGFGIKFIFCLPLAYTELSHDHL  GFNLPWGHIPIFMLVLIVAAVFHELGHAWAATSNGVTVNGFGIFILAVYPGAFTDIEAVTL  GINLPTSHLGFYALTLLICAFIHEAGHALAAVRERVRLHGFGIFVFGFYPGAFVDLNAADL  GINLPVNQLTYFFAAVLISGVVHEIGHGIAAIREQVRFNGFGIFLFIIYPGAFVDLFTTHL  GINLPVNQLTYFFTAVLISGVVHEIGHGIAAIREQVRFNGFGIFLFIIYPGAFVDLFTTHL  GVNLPISQLSYFFSAILISGVLHEVGHGVAAVRESVRFNGFGMFIFIVYPGAFVDLFTTHL  GVNLPVSQLTYFFSAILISGVIHEVGHGVAAIREQVRFNGFGIFIFIVYPGAFVDLFTTHL |
| --- | --- |

Figure S4. M16 protease family (mitochondrial processing protease, subunit beta) catalytic motif (active site shown in black)

| Schistosoma_MPPB [009650.2]  C_elegans_MPPB [Q23295]  Human_MPPB [O75439]  Chimpanzee_MPPB [Q5REK3]  Cow_MPPB [Q3SZ71]  Mouse_MPPB [Q9CXT8]  Frog_MPPB [Q0V9F0]  A_thaliana_MPPB [Q42290]  S_cerevisiae_MPPB [P10507] | ..|....|....|....|....|....|....|....|....| ....|....|....|....|...  115 125 135 145 155 185 195  GVAHFLEHMAFKGTEKRSQQSLELEVEDKGAHLNAYTSREMTV LKNSKFESSQVERERGVILREME  GTAHFLEHMAFKGTPRRTRMGLELEVENIGAHLNAYTSRESTT LLNSSLATKDIEAERGVIIREME  GTAHFLEHMAFKGTKKRSQLDLELEIENMGAHLNAYTSREQTV IQNSTLGEAEIERERGVILREMQ  GTAHFLEHMAFKGTKKRSQLDLELEIENMGAHLNAYTSREQTV IQNSTLGEAEIERERGVILREMQ  GTAHFLEHMAFKGTKKRSQLDLELEIENMGAHLNAYTSREQTV IQNSTLGEAEIERERGVILREMQ  GTAHFLEHMAFKGTKKRSQLDLELEIENMGAHLNAYTSREQTV IQNSTLGEAEIERERGVILREMQ  GTAHFLEHMAFKGTKNRSQLDLELEIENMGAHLNAYTSREQTV IQNSTLGEAEIERERGVILREMQ  GTAHFLEHMIFKGTDRRTVRALEEEIEDIGGHLNAYTSREQTT LQNSKFEEQRINRERDVILREMQ  GTAHFLEHLAFKGTQNRSQQGIELEIENIGSHLNAYTSRENTV LTKSVLDNSAIERERDVIIRESE |
| --- | --- |

Figure S5. S01 protease family catalytic motif (active site shown in black)

| A_gambiae_SP24D [Q17004]  C_elegans_LAT2 [NP_001040724]  Schistosoma_006510  Schistosoma_119130  Schistosoma_006520  Schistosoma_112090  C_elegans_TRY3 [NP_500999]  C_elegans_TRY4 [NP_508030]  C_elegans_TRY7 [NP_491910]  C_elegans_TRY6 [NP_491898]  Fruit fly_STUBBLE [Q05319]  Schistosoma_002150  Human_CFB [P00751]  Human_C2 [P06681]  Schistosoma_030350  Human_ELA1 [P08218]  Human_HGF [P14210]  Human_PLG [P00747]  Human_LPA [P08519]  Human_DF [P00746]  Human_GZMA [P12544]  Human_CMA1 [P23946]  Human_CTSG [P08311]  Human_GRZB [P10144]  Human_AZU1 [P20160]  Human_ELA2 [P08246]  Human_PRTN3 [P24158]  Schistosoma_103680  C_elegans_TRY2 [NP_501379]  C_elegans_TRY1 [NP_494910]  Human_PRSS8 [Q16651]  Human_PRSS7 [P98073]  Human_KLKB1 [P03952]  Human_F11 [P03951] | |....|....|....|  50 60  GSLIESRWVLTAAHCV  GTGTAFAWIFTILNC-  GSLVSTRAVLTAGHC-  GSLVSTRAVLTAGHC-  GSLVSSRAVLTAGHC-  GSLVSTRAVLTAGHC-  ATVIDDFWLVTAAHC-  GSIISPYHIITAAHG-  GTIVSPRHILIATHC-  GTLTSPRHILTATHCA  GALINENWIATAGHC-  GTLIAPQWILTAAHC-  GAVVSEYFVLTAAHC-  GALISDQWVLTAAHC-  GSLISAQWVMTAAHC-  GSLIANSWVLTAAHC-  GSLIKESWVLTARQC-  GTLISPEWVLTAAHC-  GTLISPEWVLTAAHC-  GVLVAEQWVLSAAHC-  GALIAKDWVLTAAHC-  GFLIRRNFVLTAAHC-  GFLVREDFVLTAAHC-  GFLIQDDFVLTAAHC-  GALIHARFVMTAASC-  ATLIAPNFVMSAAHC-  GTLIHPSFVLTAAHC-  ASLISSQWLLTAAHC-  ASILDKTHLITAAHC-  GSLIDPNFVLTAAHC-  GSLVSEQWVLSAAHC-  ASLVSSDWLVSAAHC-  GSLIGHQWVLTAAHC-  GSIIGNQWILTAAHC- | .|....|....|.  175 185  DVALLQL-QLSLP  NMIKVNGHSYPST  DIATVML-AQMVN  DIAIVML-AQMVN  DMAIITL-TNLVN  DIATVML-AQMVN  DIALLRI-SSDLS  DWAIVEV-EKRIH  DFAIVHL-YEELT  DIMIIEL-SEDVE  DLALVKL-EQPLE  DIALLRL-SEPVK  DVALIKL-KNKLK  DIALLKL-AQKVK  DIALLRL-QTPAN  DIALLKL-ANPVS  DLVLMKL-ARPAV  DIALLKL-SSPAV  DIALLKL-SRPAV  DLLLLQL-SEKAT  DLKLLQL-TEKAK  DIMLLKL-KEKAS  DIMLLQL-SRRVR  DIMLLQL-ERKAK  DLMLLQL-DREAN  DIVILQL-NGSAT  DVLLIQL-SSPAN  DYALIKI-VSPIQ  DIAILEIPYPGIE  DFAIMRI-HPPVN  DIALLQL-SRPIT  DIAMMHL-EFKVN  DIALIKL-QAPLN  DIALLKL-ETTVN | |....|....|....  355 365  VN-NGACNGDSGGP-  IERMSHASSDPRGS-  FG-QITAPGDSGGP-  FG-QLPAPGDSGGP-  SK-QIAGPGDSGGP-  FG-QITAPGDSGGP-  -YLHGTAPGDSGGP-  YSAPRTCHGDSGGG-  ASLNISLKGDSGGG-  TRTSVACPGDSGAGG  TGGQDSCQGDSGGP-  -KNKDTCAGDSGGG-  YADPNTCRGDSGGP-  QEDESPCKGESGGA-  QGGIDACQGDSGGP-  DGVICTCNGDSGGP-  KIGSGPCEGDYGGP-  AGGTDSCQGDSGGP-  ARGTDSCQGDSGGP-  SNRRDSCKGDSGGP-  RGGRDSCNGDSGSP-  RKTKSAFKGDSGGP-  RERKAAFKGDSGGP-  EIKKTSFKGDSGGP-  TRRGGICNGDGGTP-  GRQAGVCFGDSGSP-  RRKAGICFGDSGGP-  MGGKDSCQGDSGSP-  EGGIDSCQGDSGGP-  YGKIDSCQGDSGGP-  EGGKDACQGDSGGP-  EGGIDSCQGDSGGP-  EGGKDACKGDSGGP-  EGGKDACKGDSGGP- |
| --- | --- | --- | --- |

Figure S6. S54 protease family (rhomboid) catalytic motif (active site shown in black)

| Fruit fly_RHOM6 [NP_788038]  C_elegans_ROM1 [NP_498029]  Fruit fly_RHOM4 [NP_525084]  Schistosoma_020090  Human_RHBDL2 [NP_060291]  Fish_RHBDL2 [NP_957498]  Human_RHBDL1 [NP_003952]  Mouse_RHBDL1 [NP_659065]  Fish_RHBDL3 [CAM16836]  Human_RHBDL3[NP_612201]  Mouse_RHBDL3 [NP_631974]  Fruit fly_RHOM2 [NP_788450]  Fruit fly_RHOM1 [NP_52388]  Fruit fly_RHOM3 [NP_524790]  S_cerevisiae_PARL [NP_011615]  Schistosoma_032420  C_elegans_ROM5 [NP_491125]  Fruit fly_PARL [NP_523704]  Human_PARL [NP_061092]  Mouse_PARL [NP_001005767]  S_cerevisiase_RBD2 [NP_015078]  C_elegans_RHBDF1 [NP_503031]  C_elegans_RHBDF2 [NP_001076719]  Schistosoma_008620  Mouse_RHBDF2 [NP_766160]  Human_RHBDF2 [NP_078875]  Fish_RHBDF [NP_001002228]  Human_RHBDF1 [NP_071895]  Mouse_RHBDF1 [NP_034247]  Fish_RHBDL4 [NP_001017614]  Human_RHBDL4 [NP_115652]  Mouse_RHBDL4 [NP_084053] | |....|....|....|....|....|....|....|....|....|....| |....|....|....  830 840 850 860 870 890 940 950  NAWL------QPHLHLM-------GASAGVYAMLGSHVPHLVLNFSQLS-- NRNPRTSLEAHIGG—  QYAI------DPNSLLV-------GASAGVYALIFAHVANVILNWHEMP-- NDCDSVSHLAHIAG—  TSLT------SPRIFLA-------GASGGVYALITAHIATIIMNYSEME-- DQHDQIGYVAHLSG—  HSVS------DPFVLLA-------GASGGCYALIGAHLATVIMNWDIMQ-E DERTRVGFSAHFGG—  SSIF------DPLRYLV-------GASGGVYALMGGYFMNVLVNFQEMI-- EDGSPVSFAAHIAG—  SSIF------DPFSALV-------GASGGVYALMGGYFMNAIVNFREMR-- EAGLKVSFVAHIGG—  VSIT------DMRAPVV-------GGSGGVYALCSAHLANVVMNWAGMR-- ASGPQPSFMAHLAG—  VSIT------DMRAPVV-------GGSGGVYALCSAHLANVVMNWAGMR-- ASGPQPSFMAHLAG—  VSVT------DMTAPVV-------GSSGGVYALVSAHLANVVMNWSGMK-- PPCPNPSFVAHLGG—  VSVA------DMTAPVV-------GSSGGVYALVSAHLANIVMNWSGMK-- PPCPHPSFVAHLGG—  VSVA------DMTAPVV-------GSSGGVYALVSAHLANIVMNWSGMK-- PPCPHPSFVAHLGG—  TSIF------DPDVFLV-------GASGGVYALLAAHLANVLLNYHQMR-- ETAGAVSYVAHLAG—  TSVV------DSEVFLV-------GASGGVYALLAAHLANITLNYAHMK-- AKGPQVSYIAHLTG—  TSVV------DSEVFLV-------GASGGVYALLAAQLASLLLNFGQMR-- QTRPSVSYIAHMTG—  SLWYPKLARLAIVGPSL-------GASGALFGVLG---------CFSY--- LRWGSFDYAAHLGG—  SLLN—-KLLRRSTFPSL-------GASGGICAIIG---------ALSML-- SRRSALDHAAHAGG—  SIVD-KAVVRSPIRAL--------GASGAILAALT---------YTCMQ-- LRFRLFDHAAHLGG—  SVLY—-KAATSQAGMSL-------GASGAIMTLLA---------YVCTQ-- MGWKFFDHAAHLGG—  SYVG—-KVATGRYGPSL-------GASGAIMTVLA---------AVCTK-- LGWKFFDHAAHLGG—  SYVC—-KVATGRYGPSL-------GASGAIMTVLA---------AVCTK-- LGWKFFDHAAHLGG—  CLLG---KLLYPEALVA-------GASGWCFTLFAYYSFKESQIRPRTR-- ---PGSSFWGHFFG—  SAIF------VPYYPTV-------GPSSAQCGVFSSVVVELWHFRHLLD-P -----IDNWSHLFG—  SAIF------VPYNPAV-------GPSSAQCGILAAVIVECCDNRRIIKEF -----VDNWAHLFG—  SGIF------LPYQVET-------GPTGAQFALLGISLVDLIHCWQFLA-H -----IDNYANAGS—  SAIF------LPYRAEV-------GPAGSQFGLLACLFVELFQSWQLLE-R -----IDNIAHIFG—  SAIF------LPYRAEV-------GPAGSQFGLLACLFVELFQSWPLLE-R -----IDNIAHIFG—  SAIF------LPYRAEV-------GPAGSQFGILACLFVELIQSWQILA-Q -----IDNFAHISG—  SAIF------LPYRAEV-------GPAGSQFGILACLFVELFQSWQILA-R -----IDNFAHISG—  SAIF------LPYRAEV-------GPAGSQFGILACLFVELFQSWQILA-R -----IDNFAHISG—  LPIA------NRYACWVELVLIHIMNPGTSFV---GHLSGILVGLLYTT-G PYAPNTNYEQHYYGA  FPVP------NRFACWVELVAIHLFSPGTSFA---GHLAGILVGLMYTQ-G PHGRPDHYEEAPRN—  FPVP------NRFACWAELVAIHFCTPGTSFA---GHLAGILVGLMYTQ-G ADGRPVTYDATYRN— |
| --- | --- |
